# Recent, Independent and Anthropogenic Origins of *Trypanosoma cruzi* Hybrids

**DOI:** 10.1371/journal.pntd.0001363

**Published:** 2011-10-11

**Authors:** Michael D. Lewis, Martin S. Llewellyn, Matthew Yeo, Nidia Acosta, Michael W. Gaunt, Michael A. Miles

**Affiliations:** 1 Department of Pathogen Molecular Biology, Faculty of Infectious and Tropical Diseases, London School of Hygiene and Tropical Medicine, London, United Kingdom; 2 Departamento de Medicina Tropical, Instituto de Investigaciones en Ciencias de la Salud, Universidad Nacional de Asunción, Asunción, Paraguay; New York University, United States of America

## Abstract

The single celled eukaryote *Trypanosoma cruzi*, a parasite transmitted by numerous species of triatomine bug in the Americas, causes Chagas disease in humans. *T. cruzi* generally reproduces asexually and appears to have a clonal population structure. However, two of the six major circulating genetic lineages, TcV and TcVI, are TcII-TcIII inter-lineage hybrids that are frequently isolated from humans in regions where chronic Chagas disease is particularly severe. Nevertheless, a prevalent view is that hybridisation events in *T. cruzi* were evolutionarily ancient and that active recombination is of little epidemiological importance. We analysed genotypes of hybrid and non-hybrid *T. cruzi* strains for markers representing three distinct evolutionary rates: nuclear *GPI* sequences (n = 88), mitochondrial *COII*-*ND1* sequences (n = 107) and 28 polymorphic microsatellite loci (n = 35). Using Maximum Likelihood and Bayesian phylogenetic approaches we dated key evolutionary events in the *T. cruzi* clade including the emergence of hybrid lineages TcV and TcVI, which we estimated to have occurred within the last 60,000 years. We also found evidence for recent genetic exchange between TcIII and TcIV and between TcI and TcIV. These findings show that evolution of novel recombinants remains a potential epidemiological risk. The clearly distinguishable microsatellite genotypes of TcV and TcVI were highly heterozygous and displayed minimal intra-lineage diversity indicative of even earlier origins than sequence-based estimates. Natural hybrid genotypes resembled typical meiotic F1 progeny, however, evidence for mitochondrial introgression, absence of haploid forms and previous experimental crosses indicate that sexual reproduction in *T. cruzi* may involve alternatives to canonical meiosis. Overall, the data support two independent hybridisation events between TcII and TcIII and a recent, rapid spread of the hybrid progeny in domestic transmission cycles concomitant with, or as a result of, disruption of natural transmission cycles by human activities.

## Author Summary

*Trypanosoma cruzi* is a parasite that causes Chagas disease in humans, an often fatal condition affecting at least 8 million people. The clinical outcome of Chagas disease is variable, which is probably partially attributable to genetic differences between *T. cruzi* strains. Differences between *T. cruzi* strains can arise by gradual accumulation of mutations in independent lineages (clonal reproduction) or by recombination between strains, which generates new combinations of existing alleles (sexual reproduction). Although sex has been observed experimentally, and epidemiologically important *T. cruzi* subgroups (TcV TcVI) originated via hybridisation events, active recombination is considered to be extremely rare. Our aim was to determine when recombination events have occurred during the evolution of *T. cruzi*. We analysed differences within and between *T. cruzi* subgroups in DNA sequences that evolve at different speeds. We found multiple recombination events, several of which were very recent in evolutionary terms. We show that TcV and TcVI most likely originated as a result of human activities that promoted mixing between subgroups, suggesting a continuing risk for emergence and spread of new genotypes generated by sexual reproduction. We also found evidence that sexual reproduction in *T. cruzi* could involve mechanisms different from those seen in multi-cellular eukaryotes.

## Introduction

*Trypanosoma cruzi* is a single celled eukaryotic parasite, which is transmitted to vertebrate hosts via the faeces of blood-sucking triatomine bugs. It is the aetiological agent of Chagas disease in humans, which results in the death of ∼13,000 people and the loss of 649,000 disability-adjusted life years (DALYs) per year [1]. Transmission can also occur congenitally or through contaminated blood products and organs. *T. cruzi* is currently split into six genetic lineages or discrete typing units (DTUs), previously named TcI, TcIIa, IIb, IIc, IId and IIe [2], [3] but recently revised by broad consensus to TcI, TcIV, TcII, TcIII, TcV and TcVI respectively [4]. Analysis of multiple molecular markers shows that a lack of inter-lineage recombination generally preserves the individuality of each DTU [2], [5]–[7]. Recent studies of large samples of TcI and TcIII using highly variable microsatellite markers have shown that clonal population structure, may persist at an intra-lineage level [8]. However, recombination at the scale of active transmission cycles is known to occur [9] and the failure to detect it more frequently is potentially a result of high rates of inbreeding [10], gene conversion [8] or insufficient sampling of infra-populations [11].

Many pathogenic eukaryotic microorganisms have essentially clonal population structures while also having non-obligate sexual cycles, which may enable them to adapt to environmental changes [12]. Although recombination may be rare in such pathogens, it can lead to the evolution and spread of epidemiologically important traits, including those relating to virulence, transmission dynamics and drug resistance [13]. Indeed, recombination events have shaped the evolution of at least some *T. cruzi* lineages. Comparison of nucleotide sequences showed that two lineages, TcV and TcVI (which includes the genome strain CL Brener), have arisen through hybridisation between genetically distinct parents [14]–[17]. The identification of these lineages as hybrid was unequivocal because they possess fully intact alleles from two other DTUs (TcII and TcIII) that are so distinct that they could not have arisen independently. It is not clear whether TcV and TcVI are the products of independent hybridisation events [15] or a single hybridisation event followed by clonal divergence [17]. Furthermore, the resolution afforded by the genetic markers used so far has been insufficient to resolve whether these hybridisation(s) were evolutionarily ancient [14], [18] or recent events [16], [17]. The ecological circumstances of the origin of TcV and TcVI are not known. While 5S rDNA sequences suggested the TcII parent may have originated in south-western South America [17], too few TcIII isolates have been characterised sufficiently to permit similar investigation of the other putative parent.

Generation of intra-lineage TcI hybrids *in vitro* proved that the capacity for genetic exchange has been retained [19], however, there are important differences between these experimental hybrids and natural TcV and TcVI isolates [20]. Genetic characterisation of the experimental hybrids indicated they were formed by diploid-diploid fusion, forming a tetraploid intermediate, followed by genome erosion resulting in progeny with DNA contents approximately 70% higher than the parental strains [19], [20]. TcV and TcVI strains, by contrast, have DNA contents equivalent to those found in TcII and TcIII. The experimental TcI hybrids inherited >2 alleles at multiple genetic loci [19], whereas TcV and TcVI typically have only one or two alleles per locus [20]. Finally, uniparental inheritance of kDNA maxicircles occurred in both natural and experimental hybrids [16], [19]. The cytological mechanism of hybridisation in natural *T. cruzi* populations therefore remains undefined and is yet to be reconciled with that documented for *in vitro* hybrids. However, the discovery of conserved meiotic gene homologues in several basally diverging protists, including kinetoplastids, *Giardia* and *Trichomonas* implies that the common ancestor of all extant eukaryotes was capable of meiotic recombination [21], [22].

In this study molecular markers from three sequence classes (nuclear coding sequence, mitochondrial (kinetoplast maxicircle) coding sequence, and multiple microsatellite loci), each with inherently different mutation rates, were used to analyse the origins and evolution of *T. cruzi* at several overlapping levels of resolution. Recent advances in the capacity for large scale multilocus microsatellite typing [8] permitted high resolution analysis of both parental and hybrid lineages leading to novel insights into the timing, ecological circumstances and cytological mechanism of hybridisation in *T. cruzi*.

## Materials and Methods

### Samples

DNA samples for a core panel of 35 *T. cruzi* clones representing hybrid lineages TcV and TcVI and parental lineages TcII and TcIII were used for all analyses; up to 53 additional strains representing all six lineages were used in selected analyses as indicated (Tables 1 and S1). DTU-level genotypes were determined using a previously described triple-marker assay [23]. The samples were derived from diverse hosts and vectors from geographical locations representative of the distribution of each lineage.

**Table 1:** Genotypes for core samples analysed in the study.

				*GPI*		*COII-ND1*	MLMT (19 loci)	MLMT (28 loci)
				Haplotype code(s)				
Strain	DTU	Location	Host/Vector	Allele 1	Allele 2	Haplotype code	MLG code	MLG code
Tu18 cl2	TcII	Tupiza, BO	*Triatoma infestans*	Hap nG-21	Hap nG-22	Hap kCN-26	19/B01	28/B01
Rita cl5	TcII	Bahia, BR	*Homo sapiens*	Hap nG-21		Hap kCN-27	19/B02	28/B02
CBB cl2	TcII	Region IV, CL	*Homo sapiens*	Hap nG-21	Hap nG-22	Hap kCN-27	19/B03	28/B03
Pot7a cl1	TcII	Boqueron, PY	*Triatoma infestans*	Hap nG-21		Hap kCN-27	19/B04	28/B04
Pot7b cl5	TcII	Boqueron, PY	*Triatoma infestans*	Hap nG-21		Hap kCN-27	19/B04	28/B04
IVV cl4	TcII	Cuncumen, CL	*Homo sapiens*	Hap nG-21	Hap nG-22	Hap kCN-26	19/B05	28/B05
Chaco23 col4	TcII	Pr.Hayes, PY	*Triatoma infestans*	Hap nG-21	Hap nG-22	Hap kCN-25	19/B06	28/B06
Esm cl3	TcII	Bahia, BR	*Homo sapiens*	Hap nG-21		Hap kCN-27	19/B04	28/B04
T665 cl1	TcII	Pr.Hayes, PY	*Triatoma infestans*	Hap nG-22		Hap kCN-28	19/B07	28/B07
ARMA13 cl1	TcIII	Boqueron, PY	*Dasypus novemcinctus*	Hap nG-14		Hap kCN-15	19/C01	28/C01
JA2 cl2	TcIII	Amazonas, BR	*Monodelphis sp.*	Hap nG-15		Hap kCN-14	19/C02	28/C02
SABP19 cl1	TcIII	Vitor, PE	*Triatoma infestans*	Hap nG-16	Hap nG-17	Hap kCN-22	19/C03	28/C03
ARMA18 cl3	TcIII	Boqueron, PY	*Dasypus novemcinctus*	Hap nG-14	Hap nG-18	Hap kCN-13	19/C04	28/C04
CM25 cl2	TcIII	Carimaga, CO	*Dasyprocta fugilinosa*	Hap nG-15	Hap nG-19	Hap kCN-13	19/C05	28/C05
M5631 cl5	TcIII	Para, BR	*Dasypus novemcinctus*	Hap nG-15	Hap nG-20	Hap kCN-18	19/C06	28/C06
M6241 cl6	TcIII	Para, BR	*Homo sapiens*	Hap nG-15	Hap nG-20	Hap kCN-17	19/C07	28/C07
85/847 cl2	TcIII	Alto Beni, BO	*Dasypus novemcinctus*	Hap nG-15		Hap kCN-11	19/C08	28/C08
X9/3	TcIII	Pr. Hayes, PY	*Canis familiaris*	Hap nG-14		Hap kCN-22	19/C09	28/C09
X109/2	TcIII	Pr.Hayes, PY	*Canis familiaris*	Hap nG-16		Hap kCN-22	19/C10	28/C10
Sc43 cl1	TcV	Santa Cruz, BO	*Triatoma infestans*	Hap nG-22	Hap nG-23	Hap kCN-19	19/D01	28/D01
Para6 cl4	TcV	Paraguari, PY	*Triatoma infestans*	Hap nG-22	Hap nG-23	Hap kCN-20	19/D01	28/D01
92-80 cl2	TcV	Santa Cruz, BO	*Homo sapiens*	Hap nG-22	Hap nG-23	Hap kCN-21	19/D01	28/D01
Para4 cl3	TcV	Paraguari, PY	*Triatoma infestans*	Hap nG-22	Hap nG-23	Hap kCN-20	19/D01	28/D01
Chaco2 cl3	TcV	Boqueron, PY	*Triatoma infestans*	Hap nG-22	Hap nG-23	Hap kCN-20	19/D01	28/D01
PAH179 cl5	TcV	Chaco, AR	*Homo sapiens*	Hap nG-22	Hap nG-23	Hap kCN-20	19/D02	28/D02
Vinch101 cl1	TcV	Limari, CL	*Triatoma infestans*	Hap nG-22	Hap nG-23	Hap kCN-20	19/D01	28/D01
Bug2148 cl1	TcV	Rio Gr. do Sul, BR	*Triatoma infestans*	Hap nG-22	Hap nG-23	Hap kCN-20	19/D01	28/D01
CL Brener	TcVI	Rio Gr. do Sul, BR	*Triatoma infestans*	Hap nG-22	Hap nG-14	Hap kCN-24	19/E01	28/E01
VFRA1 cl1	TcVI	Francia, CL	*Triatoma infestans*	Hap nG-22	Hap nG-14	Hap kCN-24	19/E01	28/E01
Chaco17 col1	TcVI	Pr. Hayes, PY	*Triatoma infestans*	Hap nG-22	Hap nG-14	Hap kCN-24	19/E01	28/E01
Tula cl2	TcVI	Tulahuen, CL	*Homo sapiens*	Hap nG-22	Hap nG-14	Hap kCN-24	19/E02	28/E02
P251 cl7	TcVI	Cochabamba, BO	*Homo sapiens*	Hap nG-22	Hap nG-14	Hap kCN-24	19/E03	28/E03
LHVA cl4	TcVI	Chaco, AR	*Triatoma infestans*	Hap nG-22	Hap nG-14	Hap kCN-24	19/E01	28/E01
EPV20-1 cl1	TcVI	Chaco, AR	*Triatoma infestans*	Hap nG-22	Hap nG-14	Hap kCN-24	19/E02	28/E02
Chaco9 col15	TcVI	Pr. Hayes, PY	*Triatoma infestans*	Hap nG-22	Hap nG-14	Hap kCN-23	19/E01	28/E01

DTU, discrete typing unit; MLMT, multi-locus microsatellite typing; MLG, multi-locus genotype; Hap, Haplotype; BO, Bolivia; BR, Brazil; CL, Chile; PY, Paraguay; PE, Peru; CO, Colombia; AR, Argentina.

### Nucleotide sequences

Nucleotide sequences were determined for a fragment of glucose-6-phosphate isomerase (*GPI*), a single copy nuclear gene, and for a region of the kDNA maxicircle, spanning regions of two adjacent genes, cytochrome oxidase subunit II (*COII*) and NADH dehydrogenase subunit 1 (*ND1*). PCR amplification of *GPI* was performed as described previously [23]. PCR amplification reactions for *COII-ND1* contained 1× quantity NH_4_ buffer, 0.2 mM of dNTPs, 3.5 mM MgCl_2_, 1 pmol/µl of each primer, 1 Unit of *Taq* DNA polymerase (all Bioline, UK) and 10–100 ng genomic DNA using primers ND1.3A and COII.2A [16]. Amplification conditions were 94°C for 3 mins, 30 cycles of 94°C for 30 s, 58°C for 1 min, 72°C for 2 mins, followed by a final elongation step at 72°C for 10 mins.

Sequencing reactions were performed using the ABI Prism BigDye3.1 terminator cycle sequencing kit (Applied Biosystems, UK) using PCR primers and additional internal sequencing primers in some cases: for *GPI*, primer GPI.1 [19]; for *COII-ND1*, primers COII.A400 [16], COII-INTL1 (5′-CAAAAGATAATAACACTATAACAGAATC-3′) and COII-INTL2 (5′-CAAAAGATAATAACACTATAACAG-3′). For resolution of *GPI* haplotypes, PCR products were cloned using the pGEM-T Easy vector kit (Promega, UK) and inserts from up to 12 clones were sequenced. Sequences for outgroups and 43 additional *T. cruzi* strains were obtained from GenBank; accession numbers are given in Table S1.

### Phylogenetic analysis of *GPI* and *COII-ND1*

Sequences were aligned using CLUSTAL_X [24] and sequence diversity statistics calculated using DnaSP v.5 [25]. Unique haplotypes were identified and aligned with outgroup sequences to produce final alignments. Each alignment was used to reconstruct multiple alternative tree topologies in MEGA4 [26] using (a) Neighbour Joining (NJ) using Kimura's 2-parameter (K2P) [27], Tamura-Nei (TN93) [28], and maximum composite likelihood [29] substitution models and (b) Maximum Parsimony (MP) using the close neighbour interchange procedure, which generated 143 and 98 trees of equal length for *GPI* and *COII-ND1* respectively. Bootstrap support for clades was estimated using 1000 pseudo-replicate datasets. Maximum likelihood (ML) analysis was conducted for each topology using PAML v.4 [30] implementing Shimodaira-Hasegawa (SH) tests [31] to test for statistically significant differences in likelihoods between topologies. The program FINDMODEL (http://www.hiv.lanl.gov/content/sequence/findmodel/findmodel.html) was used to select the most appropriate nucleotide substitution model for each data set: GTR (REV)+gamma for *GPI* and TN93+gamma for *COII*-*ND1*. In each ML analysis a discrete gamma distribution model with four rate categories was applied to account for rate heterogeneity among sites; the gamma distribution shape parameter, α, was estimated from the data. To test for rate constancy (i.e. a molecular clock) ML analysis was performed using topologies rooted using either *Trypanosoma brucei* sequences (*GPI*) or *T. cruzi marinkellei* sequences (*COII*-*ND1*), with all branches constrained to a single rate of evolution. The resulting likelihood was compared to the unconstrained model using a likelihood ratio test (LRT).

### Estimation of divergence times

BEAST v1.5.3 [32] was used to estimate divergence times. This program implements a Bayesian Markov chain Monte Carlo (MCMC) procedure to sample the posterior probability of trees generated under a specified prior evolutionary model. One advantage of this approach is that by sampling from a large number of trees, the statistical credibility (the 95% highest posterior density (HPD) interval) associated with parameter estimates (e.g. mutation rates, divergence times) can be obtained, thereby more accurately reflecting the uncertainties of phylogenetic inference than analyses generating a single tree. For each analysis the same nucleotide substitution model as implemented in the ML analysis was used. For *GPI* we applied a strict molecular clock and for *COII*-*ND1* a relaxed clock with uncorrelated log-normally distributed rates [33]. A constant population coalescent prior was used as the demographic model. Random tree topologies were used as initial trees. To calibrate the rate estimation for *GPI* we applied a normally distributed prior on the divergence time between *T. brucei* sequences and *T. cruzi* clade sequences, i.e. the age of the root of the tree, with a mean of 100 million years ago (MYA) and SD of 2.0, according to the well supported evidence for the divergence of these groups concomitant with the separation of the African and South American land masses [34]. For *COII-ND1*, calibration was achieved in the same way but with a time applied to the *T. cruzi* – *T. cruzi marinkellei* ancestral node (the root) with a mean of 6.511 MYA and SD of 1.17, as inferred from the *GPI* results. For each data set two MCMC chains of 1×10^7^ iterations were run, with parameters logged every 1000 iterations and removal of the first 10% of states as burn-in. Log-files were checked for sufficient effective sampling sizes (ESSs) and convergence of chains on similar posterior distributions using TRACER v1.5 [35].

### Microsatellite analysis

Genotypes were obtained for the core 35 samples at 28 microsatellite loci and for an additional 46 strains at a subset of 19 microsatellite loci using previously described protocols [8], [20]; physical positions and primer details are given in Table S2. Microsatellite genotypes for some strains at some loci were from previously published datasets as indicated [8], [20], [36]. The genotypes were used to infer a measure of genetic distance between all possible pairs of strains (pairwise distance [*D*_AS_]) under the assumptions of the infinite-alleles model (IAM) [37] as previously [8]. Multi-allelic genotypes (≥3 alleles per locus), observed for 7 of 1826 genotypes, were treated as missing data. For each DTU, heterozygosity indices were calculated in ARLEQUIN 3.0 [38]. Allelic richness (A_r_) was used as a sample size-corrected measure of diversity and was calculated in FSTAT [39]. In hybrid groups TcV and TcVI, genotypes at individual loci were classed as hybrid (TcII/TcIII) or non-hybrid (TcII/TcII or TcIII/TcIII) based on the presence or absence of alleles in the TcII and TcIII parental groups. In order to minimize the possibility of null alleles affecting classification of TcV/VI genotypes, all instances of homozygosity in these samples were confirmed across three replicates. For inference of geographical relationships between TcV/VI hybrid and TcII/III parent DTUs, the genetic identity between each hybrid multilocus genotype (MLG) and all parent MLGs was calculated using 1-*D*_AS_. Then for TcV and TcVI separately, the mean identities with parental MLGs from countries with ≥2 measurements (Brazil, Bolivia, Chile and Paraguay) were calculated and compared using t-tests.

## Results

### GPI nucleotide sequence

Sequences for *GPI* were obtained for 86 *T. cruzi* strains allowing a gap-free alignment of 1038 nucleotides to be assembled. Heterozygous strains were found in all DTUs: TcI (n = 19/29), TcII (n = 4/9), TcIII (n = 8/25), TcIV (n = 1/7), TcV (n = 8/8) and TcVI (n = 8/8). A total of 34 distinct haplotypes and 58 polymorphic sites (5.59%) were identified (Tables 1 and S1). Comparing cloned and uncloned sequence reads we classified 39/247 (15.8%) as mosaics, most likely chimaeric products of PCR-mediated recombination [40], [41]. Once these were discarded two haplotypes per strain with mutually exclusive single nucleotide (SNP) patterns and compatible with the uncloned sequences were consistently identifiable.

**Figure 1:**
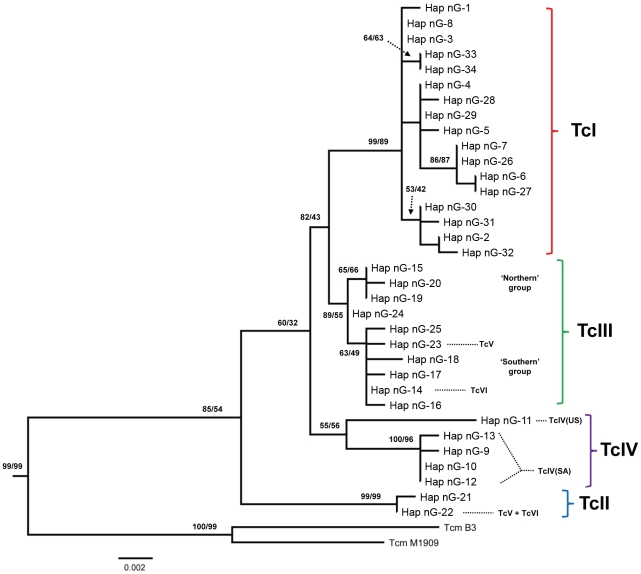
Maximum likelihood tree of *GPI* sequences (unique haplotypes). Four major clades are indicated according to corresponding *T. cruzi* lineage; TcV and TcVI haplotypes are within the parental TcII and TcIII clades from which they are derived. Where clear geographic associations were evident these are indicated: US (samples from USA); SA (samples from South America); ‘Northern’ and ‘Southern’ groups within TcIII are as defined previously [36]. Branches separating *T. cruzi* from *T. rangeli* and *T. brucei* (outgroup) are not shown. Bootstrap support values for branches (if >50%) are shown and were calculated for the equivalent nodes of the bootstrap consensus NJ/MP trees (1000 replicates). Scale units are substitutions/site.

Sequence analysis (Figure 1) identified four major clades of *T. cruzi* sequences, equating to the *T. cruzi* lineages TcI, TcII, TcIII and TcIV. Each TcV and TcVI strain had one TcII-derived and one TcIII-derived haplotype. Three of these clades (TcI, II and III) had robust bootstrap support. Sequences from North American (NA) and South American (SA) TcIV strains were clearly divergent and monophyly of these taxa was only moderately supported. Examination of mean intra-lineage pair-wise genetic distances showed that in our sample of strains the predominantly domestic lineages TcII, TcV and TcVI were much more homogeneous than strains from the predominantly sylvatic lineages TcI, TcIII and TcIV. TcIII and TcIV sequences contained fixed SNPs that were otherwise restricted to TcI or TcII, as well as multiple lineage specific polymorphisms.

All TcV and TcVI strains shared the same intact TcII haplotype (haplotype nG-22), which was also present in 5/9 TcII isolates (Tu18 cl2, CBB cl2, IVV cl4, T665 cl1 and Chaco23 col4) (Table 1). The low diversity in the TcII clade made it difficult to infer geographical associations between the present isolates and the TcII parent of TcV and TcVI but it is noteworthy that haplotype nG-22 was not found in either of the two isolates from Northern Brazil (Esm and Rita). The TcIII clade haplotype found in all TcVI strains (haplotype nG-14) was also found in 13/25 of the TcIII isolates (Table 1). A different TcIII haplotype (haplotype nG-23) was found in all TcV isolates but not in any other DTU. However, haplotype nG-23 and haplotype nG-14 were closely related, with only one distinguishing SNP (834C/G). There was evidence for some substructure within the TcIII clade, with separation between a ‘Southern’ group [36] containing all strains from Paraguay (11/11), Peru (1/1) and a subset of those from Bolivia (4/7) and a ‘Northern’ group [36], containing strains from Brazil (3/3), Colombia (2/2), Venezuela (1/1), and the remainder from Bolivia (3/7). The haplotypes from the hybrids TcV and TcVI were both unambiguously in the ‘Southern’ group (Figure 1).

### *COII-ND1* nucleotide sequence

Mitochondrial *COII-ND1* sequences were obtained for 105 *T. cruzi* strains allowing an alignment of 1118 nucleotides to be assembled. Thirty-nine unique haplotypes were identified with 198 polymorphic sites (17.7%) (Tables 1 and S1). Numerous small indels (1–3 nt) were identified, as well as some larger indels, including a large deletion of 245 nt (positions 671–915) in haplotype kCN-26, previously found in Tu18 cl2 [16], and which we also found in IVV cl4. Intra-lineage nucleotide diversities (Table S3) revealed that hybrid groups TcV and TcVI had very low genetic diversity. All TcV sequences were identical bar private SNPs in Sc43 cl1 (position 818) and 92-80 cl2 (position 1073). Likewise, all TcVI had identical sequences bar a private SNP in Chaco9 col15 (position 1025). The mean genetic distance between TcV and TcVI sequences was low (0.43%), but fixed inter-lineage SNPs were found at three positions (105, 629 and 671).

**Figure 2:**
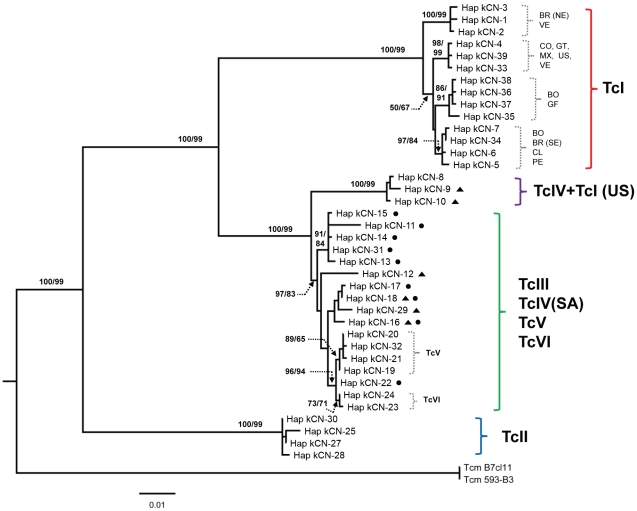
Maximum likelihood tree of *COII-ND1* sequences (unique haplotypes). Clades and subclades are indicated according to corresponding major *T. cruzi* lineage (see text). Black circles indicate TcIII haplotypes and black triangles indicate TcIV haplotypes, clearly showing paraphyly of TcIV and TcIII sequences and sharing of identical haplotypes between subsets of TcIII and TcIV samples. Where clear geographic associations were evident these are indicated using standard two letter country codes; TcIV(SA) are TcIV samples from South America. Bootstrap support values for branches (if >50%) are shown and were calculated for the equivalent nodes of the bootstrap consensus NJ/MP trees (1000 replicates). Scale units are substitutions/site.

Four major clades were recovered: (i) TcI, (ii) TcIV(NA)+three TcI(NA), (iii) TcIII+TcV+TcVI+TcIV(SA), (iv) TcII (Figure 2). Within TcI four well-supported sub-clades corresponded broadly to different geographical regions: first, all TcI sequences from Central America (5/6), Colombia (2/2) and a subset of those from Venezuela (2/3) and USA (2/5); second, sequences from South of the Amazon (Bolivia (4/7), Chile (2/2), Peru (4/4) and southern Brazil (3/3)); third, sequences from northern Brazil (5/5) and Venezuela (1/3); and fourth, sequences from French Guyana (2/2) and from a subset of isolates from Bolivia (3/7). No clear correlations between different clades and different host species or transmission cycles were apparent.

As for *GPI*, *COII*-*ND1* sequences showed clear divergence between North and South American TcIV strains. Moreover, TcIV(SA) strains were paraphyletic and interspersed between TcIII haplotypes. There were three separate instances of sharing of identical or nearly identical mitochondrial haplotypes between TcIV strains and strains from other DTUs (TcIII or TcI) for which nuclear *GPI* sequences were highly divergent (Figures 1 and 2). Such gross incongruence between mitochondrial and nuclear phylogenies is likely indicative of historical genetic exchange events resulting in mitochondrial introgression between DTUs.

The TcV and TcVI *COII*-*ND1* sequences and TcIII *COII*-*ND1* haplotype kCN-22 were more closely related to each other than to any other TcIII sequence, forming a very strongly supported monophyletic group. Haplotype kCN-22 differed from the TcV and TcVI consensus sequences at only three or one nucleotide position(s) respectively. By comparison, the next most similar TcIII isolates had fixed differences at twelve positions compared to TcVI or thirteen positions compared to TcV. Haplotype kCN-22 was found in 6/18 TcIII strains: one human isolate from southern Peru (SABP19 cl1), three were isolated from domestic dogs in the Paraguayan Chaco (X9/3, X110/8, X109/2) [42], and two were isolated from sylvatic mammals, again from the Paraguayan Chaco (Sp4, Sp16) [36], [43].

### Estimation of divergence times

For *GPI* sequences, rooted using *T. brucei*, the molecular clock model was not significantly worse than the unconstrained model (LRT p = 0.83; Table 2). Thus we used a time-calibrated strict molecular clock in the Bayesian analysis of mutation rate and divergence times for nodes in the *GPI* tree. For *COII*-*ND1* sequences, rooted using *T. cruzi marinkellei*, the molecular clock was rejected for the ML tree (LRT p = 0.032; Table 2). However, for 46/98 (46.9%) of the topologies tested the molecular clock was not rejected (p>0.05) and none of these topologies gave likelihood scores significantly worse than the ML tree (SH tests, p>0.05) under the unconstrained model. Thus, instead of a strict molecular clock we chose to apply a relaxed molecular clock model [33] permitting rate variation across the tree to be factored into the Bayesian estimation of divergence times.

**Table 2:** Molecular clock likelihood ratio tests.

Locus	Haplotypes (n)	Outgroup	Model	Clock?	Log Ln	2*ΔLn (D)	Δdf	χ^2^ p value
*GPI*	39	Unrooted	GTR+G4	No	−3236.663433			
*GPI*	39	*T. brucei*	GTR+G4	Yes	−3251.047356	28.76785	37	0.831
*COII-ND1*	40	Unrooted	TN93+G4	No	−3151.651149			
*COII-ND1*	40	*T. cruzi marinkellei*	TN93+G4	Yes	−3179.461322	55.62035	38	0.032

The mean estimated mutation rates were 3.55×10^−9^ nucleotide substitutions/site/year (95% HPD limits 2.56–4.60×10^−9^) for *GPI* and 1.94×10^−8^ (95% HPD limits 1.05–3.04×10^−8^; rate coefficient of variation = 0.34) for *COII*-*ND1*. Divergence time estimates are given in Table 3. The time of the most recent common ancestor (tMRCAs) for *T. cruzi sensu stricto* (i.e. excluding *T. cruzi marinkellei*) was estimated at 3.35 MYA (*GPI*) or 4 MYA (*COII-ND1*). For this and several other important nodes the two markers yielded similar age estimates with overlapping 95% HPD limits, including tMRCAs for TcI and TcII as well as the divergence of North American and South American TcIV. The divergence of TcI and TcIII *COII*-*ND1* sequences, estimated at 2.44 MYA (4.03–0.986), represents an estimate for the minimum age for a founding hybridisation event between TcI and TcII, assuming uniparental inheritance of the ancestral TcI maxicircle genome [17]. We considered the possibility that if TcIII-VI strains are the products of historical genetic exchange [17] then their inclusion could introduce error into the dating estimates. However, we found this not to be the case since data sets with the TcIII-VI strains included and excluded yielded virtually identical estimates of both substitution rates and divergence times (not shown). *GPI* sequences did not provide sufficient resolution to date the emergence of the hybrid DTUs TcV and TcVI but the more variable maxicircle sequences supported a very recent origin with tMRCAs estimated at 60,700 (944–126,000) years ago and 33,900 (523–91,300) years ago respectively.

**Table 3:** Divergence times for selected species and *T. cruzi* subgroups.

Alignment	Node/Group	tMRCA (MYA)	95% HPD Limits (MYA)
*GPI*	*T. brucei - T. cruzi* (root)	99.9	104 - 96
	*T. cruzi - T. rangeli*	31	39.3 - 22.9
	*T. cruzi - T. cruzi marinkellei*	6.51	8.91 - 4.33
	*T. cruzi*	3.35	4.76 - 2.06
	TcI+TcIII–TcIV	2.24	3.17 - 1.38
	TcI–TcIII	1.78	2.59 - 1.04
	TcIV(NA) - TcIV(SA)	1.65	2.48 - 0.888
	TcI	0.875	1.33 - 0.469
	TcIII	0.827	1.32 - 0.353
	TcIV(SA)	0.378	0.732 - 0.0968
	TcII	0.221	0.543 - 0.00363
*COII-ND1*	*T. cruzi - T. cruzi marinkellei* (root)	6.04	8.47 - 3.69
	*T. cruzi*	4	6.35 - 1.71
	TcI–TcIII+IV+V+VI	2.44	4.03 - 0.986
	TcIV(NA) - TcIII+IV(SA)+V+VI	0.847	1.46 - 0.317
	TcI	0.517	0.911 - 0.184
	TcII	0.148	0.303 - 0.0307
	TcV+VI+Hap22	0.138	0.26 - 0.0336
	TcV	0.0607	0.126 - 0.00944
	TcVI	0.0339	0.0913 - 0.000523

### MLMT

We applied MLMT to gain higher resolution of the genetic diversity within and between hybrid and parental lineages. The 35 core TcII, TcIII, TcV and TcVI samples were typed at 28 polymorphic microsatellite loci (28 locus-analysis; Tables 1, S1 and S4), of which eight were described previously for these strains [20]. Genotypes for additional TcI, TcIII and TcIV strains for 19 of these loci were included for comparison in some cases (19-locus analysis; Tables 1, S1 and S4). Unless additional sized peaks were observed, microsatellite profiles were considered to represent homozygous (one peak) or heterozygous (two peaks) diploid genotypes. The majority of samples gave either one or two peak sizes. Triple peak profiles were observed for CBB cl2 (TcII) at 6 loci: 6529(TA)b, 6529(CA)c, 6529(CA)a, 6529(TCC), (all linked on a 17 kb region of chromosome 6), 10101(CA)c, (chromosome 27) 11283(TA)a (chromosome 40); DNA content analysis has shown this strain is probably aneuploid [20]. The only other multi-allelic profile was found for P251 cl7 (TcVI), which had three 10101(TA)/Set0 alleles (chromosome 27).

**Figure 3:**
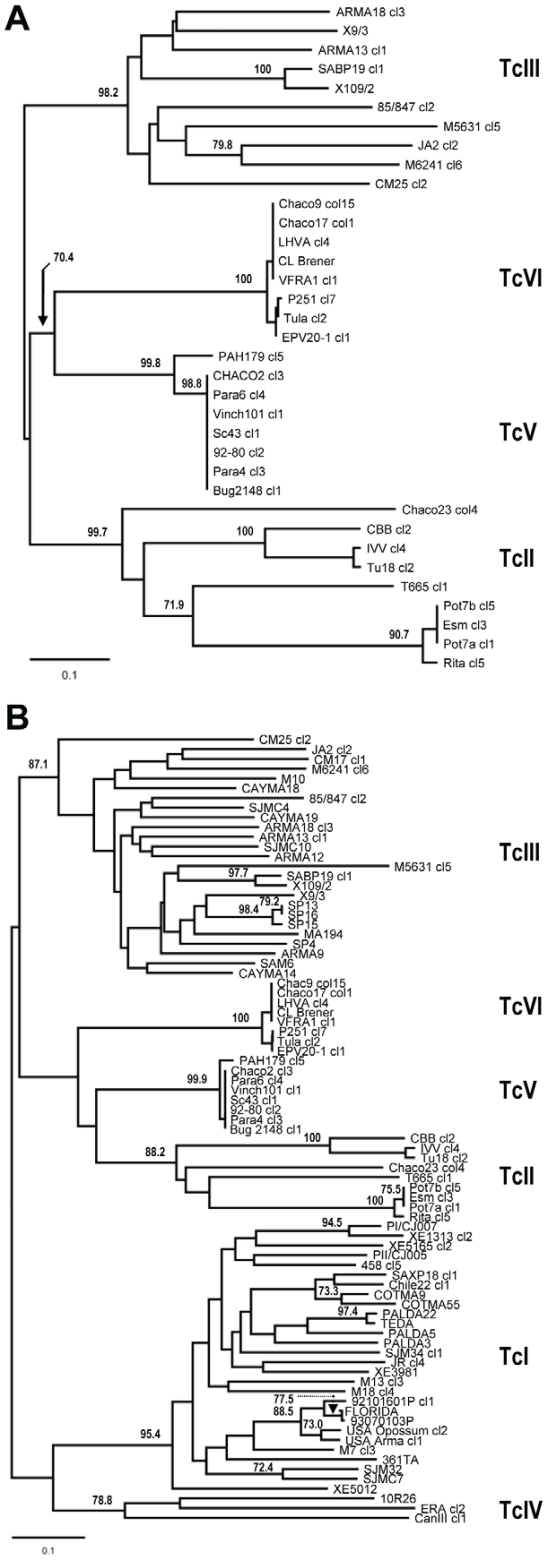
Unrooted neighbour-joining microsatellite *D*_AS_ trees showing lack of diversity within hybrid lineages TcV and TcVI. A) Tree based on 28 loci typed across 35 samples from parental (TcII, TcIII) and hybrid (TcV, TcVI) lineages. B) Tree based on 19 loci typed across 81 samples from all six major lineages TcI-VI. *D*_AS_-based bootstrap values were calculated over 1000 trees from pseudo-replicate datasets and those >70% are shown for the relevant nodes.

**Figure 4:**
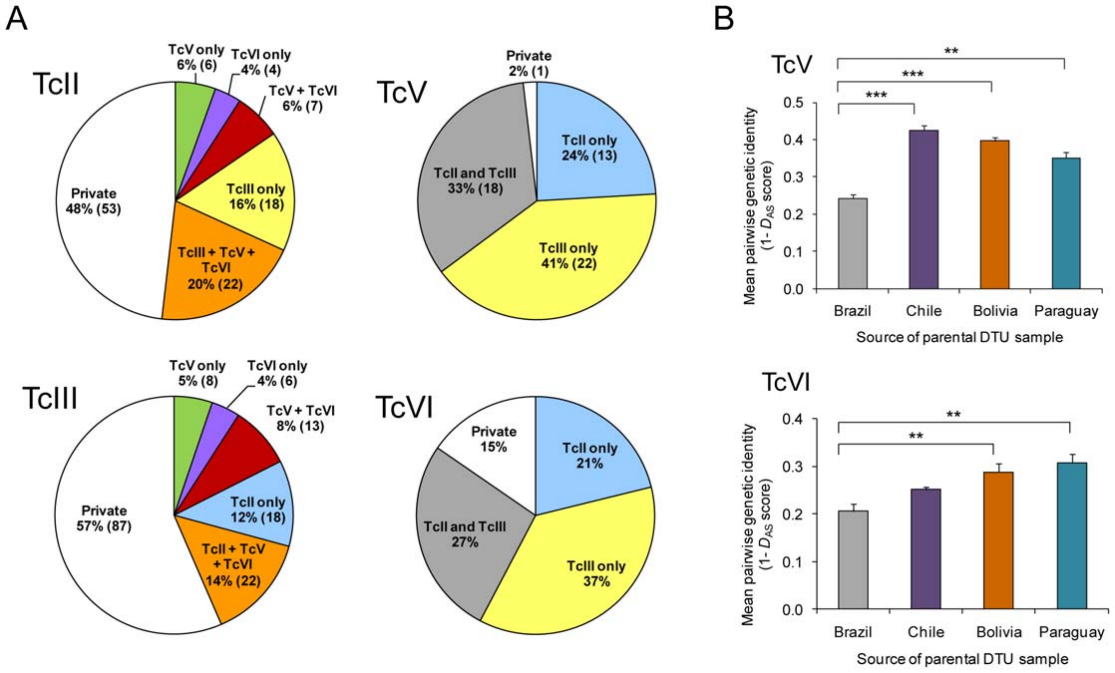
Patterns of microsatellite allelic inheritance in hybrid lineages TcV and TcVI. Based on analysis of 19 locus and 28 locus data sets combined. A) For each parental (TcII, TcIII) and hybrid (TcV, TcVI) group the charts show the proportion of microsatellite alleles that were also found in each of the other three groups and the proportion of private alleles – those not found in any of the other three groups. B) Mean pairwise genetic identities (1-*D*_AS_) between hybrid group (TcV and TcVI) MLGs and parental group (TcII and TcIII) MLGs from different geographical regions; DTU, discrete typing unit; error bars are S.E.M; brackets represent statistically significant 2-tailed t-tests with asterisks indicating: ** p<0.01, *** p<0.001.

Pairwise genetic distances (*D*_AS_) were used to reconstruct phylogenetic trees using the NJ method (Figure 3). Parental and hybrid DTUs exhibited highly dissimilar patterns of genetic diversity. Most TcII and TcIII strains had unique MLGs, with high pairwise *D*_AS_ genetic distances (>50%) and numerous private alleles (Table 4; Figure 4). In contrast TcV and TcVI were genetically uniform, with few, highly similar MLGs, low pairwise genetic distances (2.2 and 1% respectively) and far fewer private alleles (Table 4; Figure 4). Allelic richness (A_r_) provided a measure of genetic diversity independent of sample size and supported markedly lower diversity in TcV/TcVI compared to TcII/TcIII (Table 4). In the 19-locus analysis TcI, TcII, TcIII and TcIV all showed similar patterns of high genetic differentiation between strains with strong support for four monophyletic clusters (Figure 3).

**Table 4:** Multilocus microsatellite summary statistics.

Data set	DTU	N/G	Alleles	*H_o_*	*H_e_*	MNA	MNA SD	A_r_	Mean pairwise genetic distance (*D*_AS_)	Mean pairwise genetic distance (*D*_AS_) SD
28 loci	TcII	9/7	110	0.640	0.644	3.929	1.184	3.670	0.501	0.264
	TcIII	10/10	131	0.382	0.554	4.679	3.151	4.185	0.525	0.099
	TcV	8/2	54	0.817	0.450	1.929	0.466	1.919	0.022	0.039
	TcVI	8/2	52	0.786	0.424	1.857	0.525	1.821	0.010	0.009
19 loci	TcI	26/26	85	0.235	0.454	4.474	2.855	3.341	0.429	0.106
	TcIV	3/3	47	0.175	0.575	2.474	0.905	ND	0.701	0.105
	TcII	9/7	71	0.601	0.600	3.737	1.327	3.548	0.472	0.251
	TcIII	25/24	107	0.381	0.493	5.632	4.044	3.766	0.443	0.106
	TcV	8/2	35	0.836	0.449	1.842	0.375	1.842	0.007	0.011
	TcVI	8/3	36	0.842	0.456	1.895	0.459	1.895	0.014	0.014

DTU, discrete typing unit; N/G, number of samples/number of genotypes; *H_o_* observed heterozygosity; *H_e_* expected heterozygosity; MNA, mean number of alleles; A_r_ Allelic richness; ND, not determined.

Hybrid and non-hybrid DTUs were also easily distinguishable in terms of heterozygosity. TcV and TcVI were both highly heterozygous (*H*_O_ = 0.82/0.79). TcI and TcIII were far less heterozygous (*H*_O_ = 0.38/0.25). TcII displayed intermediate *H*_O_, and although this was close to overall *H*_E_, only 12/28 loci were in Hardy-Weinberg equilibrium (not shown). Identical genotypes were observed from geographically distant locations for domestic isolates from TcII, TcV and TcVI (e.g. Esm cl3 from north-eastern Brazil was identical to Pot7a cl1 and Pot7b cl5 from the Paraguayan Chaco region).

### Analysis of hybridisation

For many of the heterozygous loci in TcV and TcVI strains one of the alleles was frequently observed in TcII strains while the other allele was frequently observed in TcIII strains (Figures 4 and S1). Inheritance through hybridisation and without subsequent mutation is the most likely explanation, and was observed at too many independent loci to attribute to homoplasy. Comparison of the MLGs for TcV and TcVI also revealed fixed inter-DTU genotypic differences at 75% (21/28) of microsatellite loci and of the alleles that distinguished between TcV and TcVI 84% (37/44) were present in parental DTU strains. Coupled with the striking lack of intra-DTU diversity in both lineages this strongly suggests the two DTUs are products of separate hybridisation events.

The pairwise *D*_AS_ distances between each hybrid MLG and each TcII (28 locus analysis) and TcIII (28 and 19 locus analysis) sample were used to measure identity between members of each parental DTU and each hybrid DTU. We then used these distances to infer the approximate geographical location for the origin of TcV and TcVI by grouping hybrid-parent distances according to the country of origin of parental samples. This revealed that TcV and TcVI genotypes were most closely related to parental lineage strains from Chile and Paraguay respectively, and furthermore, TcII/III strains from Brazil were significantly less closely related to TcV/VI than those from Bolivia, Paraguay or Chile (TcV only) (Figure 4B).

The genotypes at each individual microsatellite locus in the TcV/VI isolates were examined to predict the parental origin of each allele given its presence in or absence from TcII/III isolates. In this way the predicted ancestry of hybrid alleles could be classified as (i) TcII, (ii) TcIII, (iii) TcII or TcIII, or (iv) unknown (i.e. parent not sampled or *de novo* mutation) (Figures 4A and S1). For TcV 65%, and for TcVI 58% of alleles were assigned to either TcII or TcIII; either 33% (TcV) or 27% (TcVI) were found in both TcII and TcIII; 2% (TcV) or 15% (TcVI) were found in neither parental DTU. Based on this information there was evidence of non-hybrid genotypes (i.e. both alleles derived from the same parental DTU) at three loci in TcV: 6855(TTA)(GTT) for one strain and 6789(TA) and 11283(TA)a for all strains; and two loci in TcVI 6789(TA) and 11283(TA)a for all strains; in all cases the genotypes comprised only TcIII-restricted alleles.

## Discussion

### Origin of *T. cruzi*

The subgenus *Schizotrypanum* comprises approximately half a dozen described trypanosome species, most of which evolved on the South American continent after the break-up of Gondwanaland, and are referred to as the “*T. cruzi* clade” [34], [44]. By analysing nuclear coding (*GPI*), mitochondrial coding (COII-ND1) and nuclear non-coding (microsatellites) genetic markers, we were able to resolve the evolutionary processes within this clade at multiple, over-lapping levels of resolution. *GPI* sequences had the slowest rate of evolution, estimated to have a nucleotide substitution rate of 3.55×10^−9^ substitutions/site/year, reflecting its role as a house-keeping gene. The mitochondrial locus *COII*-*ND1* had a faster evolutionary rate, estimated at 1.86×10^−8^ substitutions/site/year on average, in keeping with the generally higher rate of evolution in mtDNA than in nuclear DNA for eukaryotes [45]. Microsatellites typically have mutation rates at least three orders of magnitude higher than protein coding genes [46]. Accordingly the microsatellite loci used in this study afforded the highest resolution of intra-lineage diversity.

We estimated the dates of key events in the evolution of *T. cruzi* and related species, including divergence of *T. rangeli* and *T. cruzi* ancestral populations approximately 30 MYA, divergence of *T. cruzi s.s.* and the Chiropteran-restricted subspecies *T. cruzi marinkellei* at 6.5 – 6 MYA, and the tMRCA for *T. cruzi s.s.* at 4.0 – 3.35 MYA when the TcI and TcII lineages diverged. This date closely coincides with the formation of the land bridge between South and North America 3.5 – 3.1 MYA [47] suggesting that *T. cruzi* diversification may have been influenced by the subsequent changes in fauna known as the Great American Interchange [48]. Previous estimates for the age of *T. cruzi* range from 3–16 MYA, a date also obtained by calibration on the breakup of Gondwanaland, and 10.6 MYA calibrated using a universal rate of mutation of 1% MY^−1^ for mitochondrial *CYTB* [14]. TcIII and TcIV *GPI* sequences contained numerous SNPs that were otherwise only found in TcI or TcII, as well as lineage-specific polymorphisms. This pattern of SNPs is compatible with an ancient hybridisation event between TcI and TcII as previously suggested [17], [49]. Such reticulate evolution explains the large discrepancies between MP (based on individual sites) and NJ (based on overall genetic distance) bootstrap values for relevant nodes of the *GPI* phylogeny. The finding that the *COII*-*ND1* sequences of TcI and TcIII/IV diverged an estimated 2.44 MYA implies additional complexity because analysis of both *COII*-*ND1* [16] and *CYTB* [14] shows there is far less mitochondrial diversity both within and between TcIII and TcIV(SA) than would be expected in light of the considerable divergence observed for slower-evolving nuclear genes. This implies a mechanism acting to homogenise maxicircle sequences while nuclear sequences remain free to diverge. A partial explanation may stem from our finding of multiple instances of clearly recent mitochondrial introgression between TcIII and TcIV in South America, indicating additional recombination events involving either substantial backcrossing or kDNA exchange without exchange of nuclear material.

The sequence analysis showed clear divergence between North and South American TcIV populations, estimated at 0.85–1.65 MYA, again most likely a result of faunal migrations between the two continents. Migration of TcI from South to North America may have occurred more recently as suggested by a strong reduction in microsatellite diversity among North American TcI isolates [8]. Additionally, this study builds on evidence [16] supporting recent mitochondrial introgression between a subset of TcI and TcIV strains in North America, which share almost identical mitochondrial *COII-ND1* sequences while displaying extensive divergence for nuclear *GPI* sequences. Typing of further nuclear loci is required to determine whether such events also involved transfer of any nuclear material, but the nuclear *GPI* and microsatellite data presented here indicate that it did not.

Our estimates of substitution rates and divergence times depend on the prior assumption that *T. brucei* and *T. cruzi* diverged due to the break-up of Gondwanaland ∼100MYA. Biogeographic data indicate this is a robust assumption. For example, Stevens et al [34] showed that, with the exception of those associated with bats, *T. cruzi* clade species are found exclusively in the New World and are most closely related to a kangaroo trypanosome. This is consistent with the connection of South America to Australia via Antarctica during the late Cretaceous [50]. Molecular data show this biogeographic event also led to the evolution of the Triatominae [51]. However, the recent finding of *T. conorhini*-like and *T. vespertilionis*-like trypanosomes in two terrestrial mammals in West Africa [52] illustrates the need for caution. In this case, introduction of these trypanosomes from the New World seems a plausible explanation given the capacity for host-switching in trypanosomes and the well documented inter-continental dispersal of *T. cruzi* clade species by bats (*T. vespertilionis*, *T. dionisii*, *T. cruzi marinkellei*) and rats (*T. conorhini*) [53].

### Origin and clonal expansion of TcV and TcVI

The multilocus genotypes of the *T. cruzi* hybrid lineages TcV and TcVI fit the expectations of relatively recent hybridisation: they are highly heterozygous, exhibit minimal intra-lineage diversity and have accumulated few, if any, private alleles by *de novo* mutation. This contrasts with TcI, TcII and TcIII, which are genetically diverse, have abundant private alleles and generally have lower than expected heterozygosity [8], [36]. TcV and TcVI have undergone dramatic clonal expansion: identical MLGs were found in samples originating across a vast geographic area. Any selfing or outcrossing would have produced homozygous genotypes rather than the uniform heterozygosity observed at most loci. The minority of TcV/VI microsatellite genotypes that were homozygous can be explained by several phenomena, including inheritance of identical alleles from both parents, loss of heterozygosity (LOH) due to gene conversion, null alleles, or non-meiotic inheritance (see below).

High resolution microsatellite data allowed us to address the question of whether TcV and TcVI arose by a single event followed by clonal diversification [17] or by separate hybridisation events [15]. Our data support two independent hybridisation events between distinct TcII and TcIII strains as the most plausible scenario because they have fixed inter-lineage genotypic differences at 75% of microsatellite loci and display virtually no intra-lineage diversity. A single event leading to a hybrid population from which TcV and TcVI are the only two surviving lineages is unlikely. Firstly, the majority of microsatellite alleles that distinguish TcV and TcVI were also present in parental strains; independent inheritance is far more parsimonious than repeated homoplasy. Secondly, TcV and TcVI have distinct *GPI* and *COII*-*ND1* alleles, MLEE profiles [42], [54], [55], LSU rDNA sequences [23], [56] and MLST profiles [57]; if these differences were the result of divergence of TcV and TcVI from the same ancestral hybrid population then a higher frequency of independently derived (private) alleles at rapidly evolving microsatellite loci would be expected. Alternatively, TcV and TcVI could have evolved from distinct progeny from the same hybridisation event between heterozygous TcII and TcIII parents i.e. siblings, with different alleles inherited by the two hybrid lineages.

TcV and TcVI are almost exclusively found in domestic transmission cycles and are the predominant genotypes in some hyper-endemic regions such as the Gran Chaco area where severe forms of Chagas disease are common [42], [55], [58]–[64]. Their clonal reproductive mode and negligible genetic diversity have potentially important biological and medical implications. For instance, innate phenotypic variability for traits such as resistance to drugs used to treat Chagas disease might be lower in TcV and TcVI than in other, more diverse lineages. Equally, under certain conditions the availability of diverse alleles may result in enhanced fitness compared to either parental lineage (heterosis).

### Geographic, temporal and ecological aspects of hybridisation events

Fully intact or highly similar parental alleles identifiable in TcV and TcVI permitted phylogenetic analysis to identify TcII and TcIII strains most closely related to their actual parents. Nuclear *GPI* sequences and microsatellite genotypes both supported a significantly closer genetic relationship between TcII isolates from Chile, Paraguay and Bolivia than with TcII from Brazil. This is congruent with a study of 5S rDNA sequences [65], which linked TcV/VI to TcII from Bolivia and Chile based on the presence of an ‘invader sequence’ not found in TcII from Brazil. For the other parental DTU, TcIII, *GPI* and microsatellites supported a closer genetic relationship with TcIII from Bolivia, Paraguay and Peru than with TcIII from Brazil, Colombia or Venezuela. A previous analysis of mitochondrial *COII-ND1* sequences [16] showed that three TcIII isolates isolated from domestic dogs in the Paraguayan Chaco were most similar to TcV/VI. We identified the same *COII-ND1* sequence in a human isolate from southern Peru and in two Paraguayan Chaco sylvatic isolates. Overall, these data support the Gran Chaco and adjoining Andean valleys in south-western South America as the most probable origin of TcV and TcVI, with mitochondrial sequences pointing more specifically to the Paraguayan Chaco. Further analysis using additional strains and nuclear coding loci that afford sufficient resolution will be required to confirm this hypothesis.

This leads to the questions of how and when the hybridisation events occurred. From our mtDNA sequence analysis we conclude that the ages of the TcV and TcVI MRCAs are within approximately the last 60,000 years. At this scale, however, *COII-ND1* provided relatively low resolution, reflected by wide 95% HPD limits. The lack of allelic and genotypic diversity across so many fast-evolving microsatellite loci and the finding that the TcII and TcIII-derived genetic material appears unchanged, indicates a date towards the more recent end of the mtDNA-inferred range would be most parsimonious. *Plasmodium vivax* provides a useful comparison since it displays a similarly low level of microsatellite diversity to that found here for TcV and TcVI and is estimated to have expanded from a small founding population less than 10,000 years ago [66]. When the distribution of TcV and TcVI is considered, the data are indicative of a recent and rapid spread of what are effectively two clonal genotypes. This fits well the hypothesis [14] that the present distribution of TcV and TcVI is the product of changes in the distribution of the vector *Triatoma infestans*, which is itself thought to have spread from an initial sylvatic focus in Bolivia, across the Southern cone region and into North-eastern Brazil as a result of human-mediated dispersal and adaptation to synanthropic and domestic niches [67]–[69].

Two scenarios for TcV and TcVI origins can be considered. Firstly, limited diversity in hybrids now found in domestic cycles could result from a genetic bottleneck associated with invasion of domestic niches from more diverse, unsampled TcV/VI sylvatic populations. Records of non-domestic TcV and TcVI are, however, extremely rare. Multiple foci of wild *T. infestans* occur in the Gran Chaco, Chile and especially in highland Bolivia (reviewed in [70]), but only TcI genotypes have so far been documented [71]. Alternatively the hybrids are anthropogenic with human activities leading to a set of conditions initially promoting hybridisation between TcII and TcIII and then establishment of hybrid populations in newly available niches. Subsequent bottlenecks may have also contributed to low TcV/VI diversity. Humans had arrived in South America by 14,600 years ago and possibly earlier [72]; transmission of *T. cruzi* to humans was a frequent occurrence from at least 9,000 years ago as shown by analysis of mummified specimens [73].

Co-infection of a single host or vector would have been prerequisite for hybridisation and current distributions suggest a domestic/synanthropic setting is more likely. Like TcV and TcVI, TcII occurs predominantly in domestic transmission cycles [7], [74]–[76]. TcIII is most frequently associated with *Dasypus* armadillos but also has domestic foci involving dogs and *T. infestans* in the Gran Chaco [43], [76], [77]. The mtDNA sequences showed that the most genetically similar TcIII isolates to the hybrids were isolated mainly from domestic sources. Human activities potentially promoting co-infection include provision of habitats favourable for triatomine domiciliation [69] and practices associated with hunting [73].

When the geographic, ecological and palaeontological data are considered in the context of the extreme lack of microsatellite diversity in TcV and TcVI and the possible timescale for their emergence inferred from our data, we propose that anthropogenic hybridisation events are more plausible than invasion from a sylvatic source. TcII and TcIII are ancient lineages that would have been well adapted to sylvatic niches and so any inter-lineage hybrids would likely be out-competed. Therefore, an anthropogenic origin for the hybrids may also explain their establishment and success, which is evident from their widespread current distribution, association with domiciliated *T. infestans* and human infectivity. TcV in particular predominates in human infections in Bolivia and appears to be well-adapted to congenital transmission [78]–[81].

### Mechanism of hybridisation

*T. cruzi* laboratory hybrids produced to date are products of diploid fusion followed by genome erosion [19]. The mechanism of gene loss is incompletely characterised but does not involve a rapid return to diploidy [20]. It is yet to be determined whether such a mechanism operates in the field. Diploid fusion in *T. cruzi* is in contrast with *T. brucei* and *Leishmania major* for which laboratory crosses normally generate diploid progeny resembling standard meiotic F1 hybrids [82]–[86]. However, no haploid stages have been observed and polyploid and/or aneuploid hybrid progeny occur frequently [82], [87], [88]. There are numerous precedents for non-meiotic tetraploid to diploid transitions, for example, in *Saccharomyces cerevisiae*, as a result of repeated mitoses [89], [90], or in *Candida albicans*, in response to stress conditions [91], [92].

An expected consequence of diploid fusion followed by a 4n→2n transition is the presence of non-recombinant (non-hybrid) genotypes in 2n progeny at one third of unlinked loci, assuming that loss of chromosomal homologues from the 4n intermediate is random with respect to parentage, as observed in the *C. albicans* parasexual cycle [93]. In this context we examined the TcV and TcVI marker data but identified only 3/28 microsatellite loci with evidence for non-hybrid genotypes. Two of these loci were also homozygous and two were physically linked to other loci with hybrid, heterozygous genotypes. Therefore, LOH by gene conversion or the presence of alleles in unsampled TcII or TcIII strains are more likely explanations than uniparental inheritance. Multilocus sequence analysis has now shown evidence for relatively frequent LOH in TcV and TcVI coding sequences [57]. Some alleles may have been wrongly classified as parental due to homoplasy; however, the frequency of such errors is likely to be low. For example, of 196 alleles in TcII and TcIII only 31 were found in both DTUs, showing that independent evolution has tended to create divergent rather than convergent genotypes.

TcV and TcVI have hybrid genotypes for most markers examined in a sufficiently representative sample [16], [17], [94]–[96]. Non-hybrid (TcII/TcII or TcIII/TcIII) genotypes have been identified in TcV/VI for a small proportion of other markers, including *SL*-RNA, 24Sα rDNA (TcVI only), and *HSP70* [3], [17], [23]. Again though, LOH better explains the absence of one parental allele rather than differential loss during a 4n→2n transition, particularly since these sequences all correspond to multicopy genes, which appear more susceptible to gene conversion [97]. The CL Brener (TcVI) genome analysis gives additional support to our conclusions since equal proportions of its genome are derived from TcII as from TcIII with only 3% being classified as homozygous [98].

Thus, overall there is strong evidence that TcV and TcVI strains do not deviate significantly from the 1∶1 ratio of TcII∶TcIII alleles expected for the clonal progeny of standard meiotic F1 hybrids and so the operation of meiosis in natural cycles should not be ruled out. Scenarios involving fusion followed by either random loss of chromosomes (parasexual reduction) or by a meiotic reductive division (i.e. [4n→8n→2(4n)→4(2n)]) with independent assortment of parental chromosomes, would be expected to result in a far higher proportion of non-hybrid genotypes than is observed. Nevertheless, the lack of an observed haploid stage and recombination via diploid fusion *in vitro* suggest that the programme of sexual reproduction in *T. cruzi* may differ from canonical meiosis yet still enable production of viable diploid recombinants, for example by meiosis with constraints on chromosomal assortment [99] or one-step meiosis [100].

While the details of the cytological mechanism remain somewhat unclear, the epidemiological consequences of recent hybridisation are not: TcV and TcVI are important agents of Chagas disease that are frequently isolated from domestic transmission cycles in parts of South America where severe manifestations (e.g. megasyndromes) and congenital transmission are prevalent. It will be important to determine why the hybrids and one of their parents are well adapted to domestic transmission while the other parent has remained largely sylvatic. Modern human activities continue to disrupt sylvatic *T. cruzi* transmission cycles and may promote the emergence of novel, virulent recombinant strains.

## Supporting Information

Figure S1 **Inferred patterns of allelic inheritance in hybrid multilocus genotypes.** Unique multilocus genotypes (MLGs) for TcV (28/D01, 28/D02) and TcVI (28/E01, 28/E02, 28/E03) are shown with loci ordered in synteny based on the position of loci in the CL Brener (TcVI) reference genome sequence. Underlined loci contain fixed differences between TcV and TcVI; alleles in bold italic type indicate intra-DTU genotypic variability. Alleles are shaded according to their presence or absence among parental DTU (TcII and TcIII) samples: yellow, TcIII-restricted; blue, TcII-restricted; green, both TcII and TcIII; white, neither TcII nor TcIII. Boxed genotypes indicate putative non-hybrid genotypes.
(PDF)

Table S1 **Genotypes for all samples.**
(PDF)

Table S2 **Microsatellite locus information.**
(PDF)

Table S3 **Sequence diversity summary statistics.**
(PDF)

Table S4 **Multilocus microsatellite genotypes.**
(PDF)
