# Supplementary material for: Recent, Independent and Anthropogenic Origins of Trypanosoma cruzi Hybrids
Source: PLoS Negl Trop Dis. 2011 Oct 11;5(10):e1363. doi: 10.1371/journal.pntd.0001363 (PMC3191134; doi:10.1371/journal.pntd.0001363)
Supplement: Figure S1 — Inferred patterns of allelic inheritance in hybrid multilocus genotypes. Unique multilocus genotypes (MLGs) for TcV (28/D01, 28/D02) and TcVI (28/E01, 28/E02, 28/E03) are shown with loci ordered in synteny based on the position of loci in the CL Brener (TcVI) reference genome sequence. Underlined loci contain fixed differences between TcV and TcVI; alleles in bold italic type indicate intra-DTU genotypic variability. Alleles are shaded according to their presence or absence among parental DTU (TcII and TcIII) samples: yellow, TcIII-restricted; blue, TcII-restricted; green, both TcII and TcIII; white, neither TcII nor TcIII. Boxed genotypes indicate putative non-hybrid genotypes. (PDF) [file pntd.0001363.s001.pdf]

Locus	6529(TCC)	6529(TA)b	6529(CA)c	6529(CA)a	mclf10	6855(TG)	6855(TTA)(GTT)	11863(CA)/K368	8741(TG)	8741(TC)	8741(TA)	10101(TAA)a	10101(CA)b	10101(TA)/Set0	10101(TC)	10101(CA)c	10101(CA)a	6789(TA)	6559(TC)	10187(TA)	7093(TA)c	7093(TCC)	7093(TC)	6925(TG)b	6925(CT)	6925(TG)a	11283(TCG)	11283(TA)a
**Chromosome**	6					10		15	24			27						28	34	37	39						40	
**MLG**																												
28/D02	105	187	114	117	191	100	***227***	128	124	*180*	210	97	178	153	100	177	170	145	120	183	188	125	146	105	***178***	138	114	***125***
	111	177	120	129	185	104	***230***	128	130	*190*	226	115	170	173	104	177	174	145	120	177	190	137	156	111	***178***	146	123	***125***
28/D01	105	187	114	117	191	100	***230***	128	124	*180*	210	97	178	153	100	177	170	145	120	183	188	125	146	105	***174***	138	114	***127***
	111	177	120	129	185	104	***213***	128	130	*192*	226	115	170	173	104	177	174	145	120	177	190	137	156	111	***178***	146	123	***127***
28/E01	105	187	114	117	195	104	254	130	128	180	***210***	94	178	***153***	100	177	170	147	120	177	190	140	146	109	174	138	123	125
	111	177	120	129	185	122	213	108	136	180	***262***	115	170	***173***	106	187	172	147	120	175	190	140	170	117	178	148	138	125
28/E02	105	187	114	117	195	104	254	130	128	180	***210***	94	178	***153***	100	177	170	147	120	177	190	140	146	109	174	138	123	125
	111	177	120	129	185	122	213	108	136	180	***258***	115	170	***173***	106	187	172	147	120	175	190	140	170	117	178	148	138	125
28/E03	105	187	114	117	195	104	254	130	128	180	***210***	94	178	***153***	100	177	170	147	120	177	190	140	146	109	174	138	123	125
	111	177	120	129	185	122	213	108	136	180	***258***	115	170	***173***	106	187	172	147	120	175	190	140	170	117	178	148	138	125
														177														

[box] Non-hybrid genotypes

227 Intra-DTU differences

8741(TA) Fixed inter-DTU differences

xxx TcIII-restricted allele

xxx TcII-restricted allele

xxx TcII-TcIII shared allele

xxx Allele absent from TcII/III

Supplementary Figure S1. Inferred patterns of allelic inheritance in hybrid multilocus genotypes
