# Supplementary material for: Recent, Independent and Anthropogenic Origins of Trypanosoma cruzi Hybrids
Source: PLoS Negl Trop Dis. 2011 Oct 11;5(10):e1363. doi: 10.1371/journal.pntd.0001363 (PMC3191134; doi:10.1371/journal.pntd.0001363)
Supplement: Table S1 — Genotypes for all samples. (PDF) [file pntd.0001363.s002.pdf]

Table S1. Genotypes for all samples

Sample Set	Strain	DTU	Location	Host/Vector	*GPI* Haplotype code(s) Allele 1	Allele 2	Accession number(s) Allele 1	Allele 2	*COII-ND1* Haplotype code	Accession number	MLMT (19 loci) MLG code	MLMT (28 loci) MLG code
**Core TcII,III, V, VI**	Tu18 cl2	TcII	Tupiza, Bolivia	*Triatoma infestans*	Hap nG-21	Hap nG-22	HQ452700	HQ452701	Hap kCN-26	AF359036 [e]	19/B01	28/B01
	Rita cl5	TcII	Bahia, Brazil	*Homo sapiens*	Hap nG-21		HQ452702		Hap kCN-27	HQ604863	19/B02	28/B02
	CBB cl2	TcII	Region IV, Chile	*Homo sapiens*	Hap nG-21	Hap nG-22	HQ452703	HQ452704	Hap kCN-27	HQ604864	19/B03	28/B03
	Pot7a cl1	TcII	Boqueron, Paraguay	*Triatoma infestans*	Hap nG-21		HQ452705		Hap kCN-27	HQ604865	19/B04	28/B04
	Pot7b cl5	TcII	Boqueron, Paraguay	*Triatoma infestans*	Hap nG-21		HQ452706		Hap kCN-27	HQ604866	19/B04	28/B04
	IVV cl4	TcII	Cuncumen, Chile	*Homo sapiens*	Hap nG-21	Hap nG-22	HQ452707	HQ452708	Hap kCN-26	HQ604867	19/B05	28/B05
	Chaco23 col4	TcII	Pr. Hayes, Paraguay	*Triatoma infestans*	Hap nG-21	Hap nG-22	HQ452709	HQ452710	Hap kCN-25	HQ604868	19/B06	28/B06
	Esm cl3	TcII	Bahia, Brazil	*Homo sapiens*	Hap nG-21		HQ452711		Hap kCN-27	AF359035 [e]	19/B04	28/B04
	T665 cl1	TcII	Pr. Hayes, Paraguay	*Triatoma infestans*	Hap nG-22		HQ452712		Hap kCN-28	HQ604869	19/B07	28/B07
	ARMA13 cl1	TcIII	Boqueron, Paraguay	*Dasypus novemcinctus*	Hap nG-14		HQ452713		Hap kCN-15	HQ604870	19/C01	28/C01
	JA2 cl2	TcIII	Amazonas, Brazil	*Monodelphis sp.*	Hap nG-15		HQ452714		Hap kCN-14	HQ604871	19/C02	28/C02
	SABP19 cl1	TcIII	Vitor, Peru	*Triatoma infestans*	Hap nG-16	Hap nG-17	HQ452715	HQ452716	Hap kCN-22	HQ604872	19/C03	28/C03
	ARMA18 cl3	TcIII	Boqueron, Paraguay	*Dasypus novemcinctus*	Hap nG-14	Hap nG-18	HQ452717	HQ452718	Hap kCN-13	HQ604873	19/C04	28/C04
	CM25 cl2	TcIII	Carimaga, Colombia	*Dasyprocta fugilinosa*	Hap nG-15	Hap nG-19	GQ380671 [a]	GQ380672 [a]	Hap kCN-13	HQ604874	19/C05	28/C05
	M5631 cl5	TcIII	Marajo, Para, Brazil	*Dasypus novemcinctus*	Hap nG-15	Hap nG-20	GQ380676 [a]	GQ380677 [a]	Hap kCN-18	HQ604875	19/C06	28/C06
	M6241 cl6	TcIII	Belém, Para, Brazil	*Homo sapiens*	Hap nG-15	Hap nG-20	HQ452719	HQ452720	Hap kCN-17	AF359032 [e]	19/C07	28/C07
	85/847 cl2	TcIII	Alto Beni, Bolivia	*Dasypus novemcinctus*	Hap nG-15		GQ380661 [a]		Hap kCN-11	HQ604876	19/C08	28/C08
	X9/3	TcIII	Pr. Hayes, Paraguay	*Canis familiaris*	Hap nG-14		HQ452721		Hap kCN-22	AF359052 [e]	19/C09	28/C09
	X109/2	TcIII	Pr. Hayes, Paraguay	*Canis familiaris*	Hap nG-16		HQ452722		Hap kCN-22	AF359053 [e]	19/C10	28/C10
	Sc43 cl1	TcV	Santa Cruz, Bolivia	*Triatoma infestans*	Hap nG-22	Hap nG-23	HQ452723	HQ452724	Hap kCN-19	HQ604877	19/D01	28/D01
	Para6 cl4	TcV	Paraguari, Paraguay	*Triatoma infestans*	Hap nG-22	Hap nG-23	HQ452725	HQ452726	Hap kCN-20	HQ604878	19/D01	28/D01
	92-80 cl2	TcV	Santa Cruz, Bolivia	*Homo sapiens*	Hap nG-22	Hap nG-23	HQ452727	HQ452728	Hap kCN-21	HQ604879	19/D01	28/D01
	Para4 cl3	TcV	Paraguari, Paraguay	*Triatoma infestans*	Hap nG-22	Hap nG-23	HQ452729	HQ452730	Hap kCN-20	HQ604880	19/D01	28/D01
	Chaco2 cl3	TcV	Boqueron, Paraguay	*Triatoma infestans*	Hap nG-22	Hap nG-23	HQ452731	HQ452732	Hap kCN-20	HQ604881	19/D01	28/D01
	PAH179 cl5	TcV	Chaco, Argentina	*Homo sapiens*	Hap nG-22	Hap nG-23	HQ452733	HQ452734	Hap kCN-20	HQ604882	19/D02	28/D02
	Vinch101 cl1	TcV	Limari, Chile	*Triatoma infestans*	Hap nG-22	Hap nG-23	HQ452735	HQ452736	Hap kCN-20	HQ604883	19/D01	28/D01
	Bug2148 cl1	TcV	Rio Gr. do Sul, Brazil	*Triatoma infestans*	Hap nG-22	Hap nG-23	HQ452737	HQ452738	Hap kCN-20	HQ604884	19/D01	28/D01
	CL Brener	TcVI	Rio Gr. do Sul, Brazil	*Triatoma infestans*	Hap nG-22	Hap nG-14	HQ452739	HQ452740	Hap kCN-24	AF359041 [e]	19/E01	28/E01

	VFRA1 cl1	TcVI	Francia, Chile	*Triatoma infestans*	Hap nG-22	Hap nG-14	HQ452741	HQ452742	Hap kCN-24	HQ604885	19/E01	28/E01
	Chaco17 col1	TcVI	Pr. Hayes, Paraguay	*Triatoma infestans*	Hap nG-22	Hap nG-14	HQ452743	HQ452744	Hap kCN-24	HQ604886	19/E01	28/E01
	Tula cl2	TcVI	Tulahuen, Chile	*Homo sapiens*	Hap nG-22	Hap nG-14	HQ452745	HQ452746	Hap kCN-24	AF359042 [e]	19/E02	28/E02
	P251 cl7	TcVI	Cochabamba, Bolivia	*Homo sapiens*	Hap nG-22	Hap nG-14	HQ452747	HQ452748	Hap kCN-24	AF359048 [e]	19/E03	28/E03
	LHVA cl4	TcVI	Chaco, Argentina	*Triatoma infestans*	Hap nG-22	Hap nG-14	HQ452749	HQ452750	Hap kCN-24	HQ604887	19/E01	28/E01
	EPV20-1 cl1	TcVI	Chaco, Argentina	*Triatoma infestans*	Hap nG-22	Hap nG-14	HQ452751	HQ452752	Hap kCN-24	HQ604888	19/E02	28/E02
	Chaco9 col15	TcVI	Pr. Hayes, Paraguay	*Triatoma infestans*	Hap nG-22	Hap nG-14	HQ452753	HQ452754	Hap kCN-23	HQ604889	19/E01	28/E01
**Suppl. TcI,III,IV**	SAXP18 cl1	TcI	Majes, Peru	*Homo sapiens*	Hap nG-1		HQ452755		Hap kCN-7	HQ604890	19/X01	ND
	Chile C22 cl1	TcI	Flor de Valle, Chile	*Triatoma spinolai*	Hap nG-1	Hap nG-3	HQ452756	HQ452757	Hap kCN-6	HQ604891	19/X02	ND
	92101601P cl1	TcI	Georgia, USA	*Didelphis marsupialis*	Hap nG-3		HQ452758		Hap kCN-8	HQ604892	19/X03	ND
	JR cl4	TcI	Anzoátegui, Venezuela	*Homo sapiens*	Hap nG-3	Hap nG-5	HQ452759	HQ452760	Hap kCN-4	HQ604893	19/X04	ND
	CJ005 (PII)	TcI	Carajas, Brazil	Unidentified Bug	Hap nG-2	Hap nG-4	HQ452761	HQ452762	Hap kCN-1	HQ604894	19/X05	ND
	CJ007 (PI)	TcI	Carajas, Brazil	*Didelphis marsupialis*	Hap nG-6	Hap nG-7	HQ452763	HQ452764	Hap kCN-2	HQ604895	19/X06	ND
	B187 cl10	TcI	Pará, Brazil	*Didephis marsupialis*	Hap nG-2	Hap nG-8	HQ452765	HQ452766	Hap kCN-3	HQ604896	ND	ND
	C8 cl1	TcI	La Paz, Bolivia	*Triatoma infestans*	Hap nG-1	Hap nG-2	HQ452767	HQ452768	Hap kCN-5	HQ604897	ND	ND
	X10/1	TcI	Pará, Brazil	*Homo sapiens*	Hap nG-3	Hap nG-4	HQ452769	HQ452770	Hap kCN-1	AF359013 [e]	ND	ND
	FLORIDA	TcI	Florida, USA	*Triatoma sanguisuga*	Hap nG-3		HQ452771		Hap kCN-8	AF359010 [e]	19/X07	ND
	M7	TcI	Barinas, Venezuela	*Didelphis marsupialis*	Hap nG-7	Hap nG-6	HQ452772	HQ452773	ND		19/X08	ND
	Xe1313	TcI	Carajas, Brazil	*Philander opossum*	Hap nG-26	Hap nG-27	HQ452774	HQ452775	ND		19/X09	ND
	Xe3981	TcI	Para, Brazil	*Didelphis marsupialis*	Hap nG-28	Hap nG-29	HQ452776	HQ452777	ND		19/X10	ND
	Sjm34	TcI	Beni, Bolivia	*Didelphis marsupialis*	Hap nG-2	Hap nG-29	HQ452778	HQ452779	ND		19/X11	ND
	Xe5012	TcI	Para, Brazil	*Didelphis marsupialis*	Hap nG-29	Hap nG-2	HQ452780	HQ452781	ND		19/X12	ND
	COTMA9	TcI	Cotopachi, Bolivia	*Phyllotis ocilae*	Hap nG-30	Hap nG-1	HQ452782	HQ452783	ND		19/X13	ND
	PALDA22	TcI	Chaco, Argentina	*Didelphis albiventris*	Hap nG-30	Hap nG-34	HQ452784	HQ452785	ND		19/X14	ND
	COTMA55	TcI	Cotopachi, Bolivia	*Phyllotis ocilae*	Hap nG-30	Hap nG-1	HQ452786	HQ452787	ND		19/X15	ND
	458	TcI	Bajo Calima, Colombia	*Potus flavus*	Hap nG-31	Hap nG-32	HQ452788	HQ452789	ND		19/X16	ND
	PALDA3	TcI	Chaco, Argentina	*Didelphis albiventris*	Hap nG-2		HQ452790		ND		19/X17	ND
	TEDA	TcI	Chaco, Argentina	*Didelphis albiventris*	Hap nG-2	Hap nG-34	HQ452791	HQ452792	ND		19/X18	ND
	361ta	TcI	Guadual, Colombia	*Didelphis marsupialis*	Hap nG-3		HQ452793		ND		19/X19	ND
	M13	TcI	Barinas, Venezuela	*Didelphis marsupialis*	Hap nG-3		HQ452794		ND		19/X20	ND
	M18	TcI	Barinas, Venezuela	*Didelphis marsupialis*	Hap nG-3		HQ452795		ND		19/X21	ND
	93070103P	TcI	Georgia. USA	*Didelphis marsupialis*	Hap nG-3		HQ452796		Hap kCN-8	HQ604898	19/X07	ND
	PALDA5	TcI	Chaco, Argentina	*Didelphis albiventris*	Hap nG-3	Hap nG-34	HQ452797	HQ452798	ND		19/X23	ND

	Sjm32	TcI	Beni, Bolivia	*Philander opossum*	Hap nG-3		HQ452799		ND		19/X24	ND
	Sjmc7	TcI	Beni, Bolivia	*Scuireus spadiceus*	Hap nG-33	Hap nG-3	HQ452800	HQ452801	ND		19/X25	ND
	Xe5165	TcI	Para, Brazil	*Didelphis marsupialis*	Hap nG-3		HQ452802		ND		19/X26	ND
	USA Arma cl1	TcI	Louisiana, USA	*Dasypus novemcinctus*	ND	ND			Hap kCN-39	HQ604899	19/X22	ND
	USA Opossum cl2	TcI	Louisiana, USA	*Didelphis marsupialis*	ND	ND			Hap kCN-39	HQ604900	19/X27	ND
	CM17	TcIII	Carimaga, Colombia	*Dasypus spp*	Hap nG-15		HQ452803		Hap kCN-16	AF359033 [e]	19/C11	ND
	m10	TcIII	Barinas, Venezuela	*Dasypus novemcinctus*	Hap nG-24	Hap nG-15	GQ380673 [a]	GQ380674 [a]	ND		19/C12	ND
	cayma19	TcIII	Santa Cruz, Bolivia	*Dasypus novemcinctus*	Hap nG-15		GQ380667 [a]		ND		19/C13	ND
	Ma194	TcIII	Boqueron, Paraguay	*Chaetophractus vellorosus*	Hap nG-14		GQ380683 [a]		ND		19/C14	ND
	Sp4	TcIII	San Pedro, Paraguay	*Monodelphis domestica*	Hap nG-14		GQ380688 [a]		Hap kCN-22	HQ604901	19/C15	ND
	Sjmc4	TcIII	Beni, Bolivia	*Dasypus novemcinctus*	Hap nG-15		GQ380663 [a]		Hap kCN-31	HQ604902	19/C16	ND
	Sjmc10	TcIII	Beni, Bolivia	*Dasypus novemcinctus*	Hap nG-15		GQ380662 [a]		Hap kCN-31	HQ604903	19/C17	ND
	Sam6	TcIII	Santa Cruz, Bolivia	*Dasypus novemcinctus*	Hap nG-18	Hap nG-14	GQ380669 [a]	GQ380670 [a]	Hap kCN-31	HQ604904	19/C18	ND
	Cayma14	TcIII	Santa Cruz, Bolivia	*Dasypus novemcinctus*	Hap nG-25	Hap nG-14	GQ380664 [a]	GQ380665 [a]	Hap kCN-31	HQ604905	19/C19	ND
	Arma12	TcIII	Boqueron, Paraguay	*Dasypus novemcinctus*	Hap nG-14		GQ380680 [a]		ND		19/C20	ND
	Arma9	TcIII	Boqueron, Paraguay	*Dasypus novemcinctus*	Hap nG-14		GQ380682 [a]		ND		19/C21	ND
	Cayma18	TcIII	Santa Cruz, Bolivia	*Dasypus novemcinctus*	Hap nG-14		GQ380666 [a]		ND		19/C22	ND
	Sp13	TcIII	San Pedro, Paraguay	*Dasypus novemcinctus*	Hap nG-14		GQ380685 [a]		ND		19/C23	ND
	Sp15	TcIII	San Pedro, Paraguay	*Dasypus novemcinctus*	Hap nG-14		GQ380686 [a]		ND		19/C24	ND
	Sp16	TcIII	San Pedro, Paraguay	*Dasypus novemcinctus*	Hap nG-14		GQ380687 [a]		Hap kCN-22	HQ604906	19/C23	ND
	CanIII cl1	TcIV	Belém, Brazil	*Homo sapiens*	Hap nG-9		HQ452804		Hap kCN-12	AF359030 [e]	19/A01	ND
	10R26	TcIV	Santa Cruz, Bolivia	*Aotus sp.*	Hap nG-10		HQ452805		Hap kCN-16	HQ604907	19/A02	ND
	ERA cl2	TcIV	Anzoátegui, Venezuela	*Homo sapiens*	Hap nG-10		HQ452806		Hap kCN-16	HQ604908	19/A03	ND
	92122102R	TcIV	Georgia, USA	*Procyon lotor*	Hap nG-11		HQ452807		Hap kCN-10	HQ604909	ND	ND
	StC10R cl1	TcIV	Georgia, USA	*Procyon lotor*	Hap nG-11		HQ452808		Hap kCN-9	HQ604910	ND	ND
	Saimiri3 cl1	TcIV	Venezuela	*Saimiri sciureus*	Hap nG-12	Hap nG-13	HQ452809	HQ452810	Hap kCN-18	HQ604911	ND	ND
	X10610 cl5	TcIV	Guárico, Venezuela	*Homo sapiens*	Hap nG-10		HQ452811		Hap kCN-16	HQ604912	ND	ND
**Outgroups**	*T. cruzi marinkellei* B7	NA	Bahia, Brazil	*Phyllostomus discolor*	ND		ND		Tcm B7cl11	AF359055 [e]	ND	ND
	T. cruzi marinkellei 593/B3	NA	Bahia, Brazil	*Phyllostomus discolor*	Tcm B3		AY484484 [b]		Tcm 593-B3	AF359054 [e]	ND	ND
	T. cruzi marinkellei M1909	NA	Caracas, Venezuela	*Phyllostomus discolor*	Tcm M1909		HQ452812		ND		ND	ND
	T. rangeli RGB	NA	Caracas, Venezuela	*Canis familiaris*	Tr RGB		AY484486 [b]		ND		ND	ND
	T. brucei TREU927	NA	Kenya	*Glossina palpalis*	Tb 927		AL929603 [c]		ND		ND	ND

	T. brucei 427	NA	Uganda	*Ovis aires*	Tb 427		X15540 [d]	ND		ND	ND
**Genbank *COII-ND1***	MAV	TcI	Venezuela	*Homo sapiens*	ND	ND		Hap kCN-1	AF359016 [e]	ND	ND
	X10/4	TcI	Belém, Brazil	*Homo sapiens*	ND	ND		Hap kCN-1	EU302222 [f]	ND	ND
	CUTIA	TcI	Espiritu Santo, Brazil	*Dasyprocta sp*	ND	ND		Hap kCN-6	AF359021 [e]	ND	ND
	V121	TcI	Region II, Chile	*Triatoma infestans*	ND	ND		Hap kCN-6	AF359018 [e]	ND	ND
	P209	TcI	Sucre, Bolivia	*Homo sapiens*	ND	ND		Hap kCN-6	AF359023 [e]	ND	ND
	CEPA_EP	TcI	Unknown	Unknown	ND	ND		Hap kCN-33	AF359011 [e]	ND	ND
	Teh	TcI	Mexico	Unidentified bug	ND	ND		Hap kCN-33	AF359009 [e]	ND	ND
	VinC6	TcI	Cundinamarca, Colombia	*Didelphis marsupialis*	ND	ND		Hap kCN-33	AF359012 [e]	ND	ND
	OPS21	TcI	Cojedes, Venezuela	*Homo sapiens*	ND	ND		Hap kCN-33	AF359020 [e]	ND	ND
	171	TcI	Guatemala	*Triatoma dimidiata*	ND	ND		Hap kCN-33	EU302205 [f]	ND	ND
	H15	TcI	Guatemala	*Homo sapiens*	ND	ND		Hap kCN-33	EU302206 [f]	ND	ND
	SJA-3	TcI	Guatemala	*Triatoma dimidiata*	ND	ND		Hap kCN-33	EU302207 [f]	ND	ND
	SJA-8	TcI	Guatemala	*Triatoma dimidiata*	ND	ND		Hap kCN-33	EU302208 [f]	ND	ND
	St5	TcI	Guatemala	*Triatoma dimidiata*	ND	ND		Hap kCN-33	EU302209 [f]	ND	ND
	Colombia	TcI	Colombia	*Homo sapiens*	ND	ND		Hap kCN-33	EU302213 [f]	ND	ND
	T-P	TcI	Peru	*Triatoma infestans*	ND	ND		Hap kCN-34	EU302215 [f]	ND	ND
	ACP-P	TcI	Peru	*Homo sapiens*	ND	ND		Hap kCN-34	EU302214 [f]	ND	ND
	Esquilo	TcI	São Paulo, Brazil	*Sciurus aestuans*	ND	ND		Hap kCN-34	AF359028 [e]	ND	ND
	P0AC	TcI	Bolivia	*Homo sapiens*	ND	ND		Hap kCN-34	AF359027 [e]	ND	ND
	SO34 cl4	TcI	Potosi, Bolivia	*Triatoma infestans*	ND	ND		Hap kCN-34	AF359025 [e]	ND	ND
	CUICA cl1	TcI	São Paulo, Brazil	*Philander opossum*	ND	ND		Hap kCN-34	AF359024 [e]	ND	ND
	SABP3	TcI	Vitor, Peru	*Triatoma infestans*	ND	ND		Hap kCN-34	AF359017 [e]	ND	ND
	SC13	TcI	Unknown	Unknown	ND	ND		Hap kCN-35	AF359029 [e]	ND	ND
	A80	TcI	Montsinery, Fr. Guyana	*Didelphis marsupialis*	ND	ND		Hap kCN-36	AF359014 [e]	ND	ND
	A92	TcI	Acarouany, Fr.Guyana	*Didelphis marsupialis*	ND	ND		Hap kCN-36	AF359015 [e]	ND	ND
	85/818	TcI	Alto Beni, Bolivia	*Didelphis marsupialis*	ND	ND		Hap kCN-37	AF359026 [e]	ND	ND
	Ab3-4	TcI	Bolivia	*Triatoma infestans*	ND	ND		Hap kCN-38	EU302216 [f]	ND	ND
	26 79	TcI	Santa Cruz, Bolivia	*Triatoma sordida*	ND	ND		Hap kCN-38	AF359019 [e]	ND	ND
	133 79	TcI	Santa Cruz, Bolivia	*Homo sapiens*	ND	ND		Hap kCN-38	AF359022 [e]	ND	ND
	MVB	TcII	Region IV, Chile	*Homo sapiens*	ND	ND		Hap kCN-26	AF359051 [e]	ND	ND
	X-300	TcII	Paraiba, Brazil	*Homo sapiens*	ND	ND		Hap kCN-27	AF359049 [e]	ND	ND
	MSC2	TcII	Brasilia, Brazil	*Homo sapiens*	ND	ND		Hap kCN-27	AF359038 [e]	ND	ND

MCV	TcII	Region IV, Chile	*Homo sapiens*	ND	ND	Hap kCN-30	AF359050 [e]	ND	ND
X110/8	TcIII	Pr. Hayes, Paraguay	*Canis familiaris*	ND	ND	Hap kCN-22	AF359034 [e]	ND	ND
Ep255	TcIV	Meta, Colmobia	*Rhodnius prolixus*	ND	ND	Hap kCN-29	AF359031 [e]	ND	ND
BRJ	TcIV	Guatemala	*Homo sapiens*	ND	ND	Hap kCN-16	EU302217 [f]	ND	ND
SO3 cl5	TcV	Potosi, Bolivia	*Triatoma infestans*	ND	ND	Hap kCN-20	AF359039 [e]	ND	ND
86-1	TcV	Santa Cruz, Bolivia	Unknown	ND	ND	Hap kCN-20	AF359043 [e]	ND	ND
Ab3-10	TcV	Bolivia	*Triatoma infestans*	ND	ND	Hap kCN-20	EU302218 [f]	ND	ND
EPP	TcV	Region I, Chile	*Homo sapiens*	ND	ND	Hap kCN-32	AF359044 [e]	ND	ND
86/2036	TcVI	Bolivia	*Didelphis marsupialis*	ND	ND	Hap kCN-24	AF359047 [e]	ND	ND
VMV4	TcVI	Unknown	Unknown	ND	ND	Hap kCN-24	AF359045 [e]	ND	ND
P63 cl1	TcVI	Pr. Hayes, Paraguay	*Triatoma infestans*	ND	ND	Hap kCN-24	AF359046 [e]	ND	ND

DTU, discrete typing unit; MLMT, multilocus microsatellite typing; MLG, multilocus genotype; Hap, haplotype; ND, not determined

[a], [1]; [b], [2];c, [3]; d, [4]; e, [5]; f, [6].

1. Llewellyn MS, Lewis MD, Acosta N, Yeo M, Carrasco HJ, et al. (2009) *Trypanosoma cruzi* IIc: Phylogenetic and Phylogeographic Insights from Sequence and Microsatellite Analysis and Potential Impact on Emergent Chagas Disease. PLoS Negl Trop Dis 3: e510.

2. Broutin H, Tarrieu F, Tibayrenc M, Oury B, Barnabé C (2006) Phylogenetic analysis of the glucose-6-phosphate isomerase gene in *Trypanosoma cruzi*. Exp Parasitol 113: 1-7.

3. Hall N, Berriman M, Lennard NJ, Harris BR, Hertz-Fowler C, et al. (2003) The DNA sequence of chromosome I of an African trypanosome: gene content, chromosome organisation, recombination and polymorphism. Nucleic Acids Res 31: 4864-4873.

4. Marchand M, Kooystra U, Wierenga RK, Lambeir A-M, Van Beeumen J, et al. (1989) Glucosephosphate isomerase from *Trypanosoma brucei*. Eur J Biochem 184: 455-464.

5. Machado CA, Ayala FJ (2001) Nucleotide sequences provide evidence of genetic exchange among distantly related lineages of *Trypanosoma cruzi*. Proc Natl Acad Sci USA 98: 7396-7401.

6. Iwagami M, Higo H, Miura S, Yanagi T, Tada I, et al. (2007) Molecular phylogeny of *Trypanosoma cruzi* from Central America (Guatemala) and a comparison with South American strains. Parasitol Res 102: 129-134.
