# Supplementary material for: Recent, Independent and Anthropogenic Origins of Trypanosoma cruzi Hybrids
Source: PLoS Negl Trop Dis. 2011 Oct 11;5(10):e1363. doi: 10.1371/journal.pntd.0001363 (PMC3191134; doi:10.1371/journal.pntd.0001363)
Supplement: Table S2 — Microsatellite locus information. (PDF) [file pntd.0001363.s003.pdf]

Table S2. Microsatellite locus information

Locus	Reference chromosome [a]	Repeat type	Physical position (kb) [a]	Primer 1 (5'-3')	Primer 2 (5'-3')
6529(TCC)	6-S	(TCC)n	29916.429	ATTCCTTGTCGGTGAACACC	GCCCTCCGTTTATTCCTCTC
6529(TA)b	6-S	(TA)n	29916.841	TGAAGGAGATTCTCTGCGGT	CTCTCATCTTTTGTTGTGTCCG
6529(CA)c	6-S	(CA)n	29920.007	GCCTCTATGTCTGTGCCCAT	CGTCTTTTCTCGGGTGTTTC
6529(CA)a	6-S	(CA)n	29933.413	TGTGAAATGATTTGACCCGA	AGAGTCACGCCGCAAAGTAT
MCLF10 [b]	6-S	(CA)n	30127.119	GCGTAGCGATTCATTTCC	ATCCGCTACCACTATCCAC
6855(TG)	10-P	(TG)n	404.483	TTGCGTGGTTGTTTGTGC	GAGAAGAGGGGGAGGAAGAA
6855(TTA)(GTT)	10-P	(TTA)n…(GTT)n	407.438	GAGGTGATGACGATAAAATTGG	GTCTTTCCGCATATCCGAGA
11863(CA)/K368 [c]	15-P	(CA)n	2856.622	AGTTGACATCCCCAAGCAAG	CCCTGATGCTGCAGACTCTT
8741(TG)	24-P	(CT)n…(TG)n	8873.863	AGAAGAGAGTCCGGAGTTTTC	TGTGCGCCAAGAGTTCTAAA
8741(TC)	24-P	(TC)n	8903.919	GCCATGTTTCCTTCACCAAC	AGGTGTTCCCCTCTTTGGAT
8741(TA)	24-P	(TA)n	8920.522	TGTAACGGTAGGTCTCAATTCG	TTGCACTTGTGTATCTCGCC
10101(TAA)a	27-S	(TAA)n	10949.242	CCGCGGTAGAAGAACCATAA	TGCGTATTCACGACGAGAAG
10101(CA)b	27-S	(CA)n	10963.959	ACCCAGAGGGGAGAAAAAGA	TTTACGGTTGGTTCGTGTGA
10101(TA)/Set0 [b]	27-S	(TA)n	10995.038	CCTCTGCGCACACATTCATT	CCGTTCTTCATCACCATCCT
10101(TC)	27-S	(TC)n	10997.7	CGTACGACGTGGACACAAAC	ACAAGTGGGTGAGCCAAAAG
10101(CA)c	27-S	(CA)n	11035.78	GTGTCGTTGCTCCCAAACTC	AAACTTGCCAAATGTGAGGG
10101(CA)a	27-S	(CA)n	11036.757	GTCGCCATCATGTACAAACG	CTGTTGGCGAATGGTCATAA
6789(TA)	28-P	(TA)n	11833.37	GACATGGAAAAAGGTAAGTGC	GAGGACGTCATGATGATTGG
6559(TC)	34-P	(TC)n	17976.143	CGCTCTCAAAGGCACCTTAC	ATATGGACGCGTAGGAGTGC
10187(TA)	37-P	(TA)n	21897.051	AGAAAAAGGTTTACAACGAGCG	CGATGGAGAACGTGAAACAA
7093(TA)c	39-S	(TA)n	23338.719	CGTGTGCACAGGAGAGAAAA	CGTTTGGAGGAGGATTGAGA
7093(TCC)	39-S	(TCC)n	23348.315	AGACGTTCATATTCGCAGCC	AGCCACATCCACATTTCCTC
7093(TC)	39-S	(TC)n	23392.52	CCAACATTCAACAAGGGAAA	GCATGAATATTGCCGGATCT
6925(TG)b	39-S	(TG)n	23460.356	GAAACGCACTCACCCACAC	GGTAGCAACGCCAAACTTTC
6925(CT)	39-S	(CT)n	23501.037	CATCAAGGAAAAACGGAGGA	CGGTACCACCTCAAGGAAAG
6925(TG)a	39-S	(TG)n	23531.787	TCGTTCTCTTTACGCTTGCA	TAGCAGCACCAAACAAAACG
11283(TCG)	40-P	(TCG)n	26042.76	ACCACCAGGAGGACATGAAG	TGTACACGGAACAGCGAAG
11283(TA)a	40-P	(TA)n	26114.044	CCAGTATTCCTCCCCCCTAA	CGTCTTGTTGATTTCCCCTT

[a] Based on *T. cruzi* CL Brener genome v.2009-09-10 hosted on http://tritryp.org
[b] Previously described [1]
[c] Previously described [2]

1. Oliveira RP, Broude NE, Macedo AM, Cantor CR, Smith CL, et al. (1998) Probing the genetic population structure of *Trypanosoma cruzi* with polymorphic microsatellites. Proc Natl Acad Sci USA 95: 3776-3780.

2. Gaunt MW, Yeo M, Frame IA, Stothard JR, Carrasco HJ, et al. (2003) Mechanism of genetic exchange in American trypanosomes. Nature 421: 936-939.
