# Supplementary material for: Recent, Independent and Anthropogenic Origins of Trypanosoma cruzi Hybrids
Source: PLoS Negl Trop Dis. 2011 Oct 11;5(10):e1363. doi: 10.1371/journal.pntd.0001363 (PMC3191134; doi:10.1371/journal.pntd.0001363)
Supplement: Table S3 — Sequence diversity summary statistics. (PDF) [file pntd.0001363.s004.pdf]

Table S4. Sequence diversity summary statistics

Locus	Group	N [a]	L [b]	S [c]	k [d]	h [e]	Hd [f]	Hd SD	π [g]	π SD
GPI	*T. cruzi*	172	1038	58	10.502	34	0.918	0.009	0.0101	0.0005
	TcI	58	1038	13	1.969	17	0.840	0.039	0.0019	0.0002
	TcII	18	1038	1	0.471	2	0.471	0.082	0.0005	0.0001
	TcIII	50	1038	9	1.441	9	0.691	0.048	0.0014	0.0001
	TcIV	14	1038	15	6.209	5	0.758	0.084	0.0060	0.0012
	TcV	16	1038	17	9.067	2	0.533	0.046	0.0087	0.0008
	TcVI	16	1038	18	9.6	2	0.533	0.046	0.0093	0.0008
COII-ND1	*T. cruzi*	102 [h]	1117 (1094)	195	51.788	38	0.957	0.007	0.0473	0.0023
	TcI	42	1114 (1109)	112	20.129	15	0.900	0.026	0.0182	0.0045
	TcII	10 [h]	1116 (1114)	7	1.556	4	0.533	0.180	0.0014	0.0006
	TcIII	18	1115 (1100)	26	6.948	9	0.856	0.062	0.0063	0.0009
	TcIV	9	1114 (1109)	45	15.444	6	0.833	0.127	0.0139	0.0037
	TcV	12	1114	3	0.5	4	0.029	0.170	0.0005	0.0002
	TcVI	11	1114	1	0.182	2	0.182	0.144	0.0002	0.0001

[a] Number of sequences; 2 per sample for *GPI* (diploid), 1 per sample for *COII-ND1* (haploid)
[b] Length of sequence (excluding sites with alignment gaps)
[c] Number of variable sites
[d] Mean number of pairwise nucleotide differences
[e] Number of haplotypes
[f] Haplotype diversity
[g] Per site nucleotide diversity
[h] Three samples (haplotype 26) excluded due to large deletion
