# Supplementary material for: Recent, Independent and Anthropogenic Origins of Trypanosoma cruzi Hybrids
Source: PLoS Negl Trop Dis. 2011 Oct 11;5(10):e1363. doi: 10.1371/journal.pntd.0001363 (PMC3191134; doi:10.1371/journal.pntd.0001363)
Supplement: Table S4 — Multilocus microsatellite genotypes. (PDF) [file pntd.0001363.s005.pdf]

Table S6. Multilocus microsatellite genotypes

Group	Sample	MLG[a] codes (19 locus; 28 locus)	6529(TCC)	6529(TA)b	6529(CA)c	6529(CA)a	mclf10	6855(TG)	6855(TTA)(GTT)	11863(CA)/K368	8741(TG)	8741(TC)	8741(TA)	10101(TAA)a	10101(CA)b	10101(TA)/Set0	10101(TC)	10101(CA)c	10101(CA)a	6789(TA)	6559(TC)	10187(TA)	7093(TA)c	7093(TCC)	7093(TC)	6925(TG)b	6925(CT)	6925(TG)a	11283(TCG)	11283(TA)a
Core TcII[b]	Rita cl5	19/B02; 28/B02	102	177	120	117	183	102	219	114	134	178	234	121	170	165	108	177	166	153	116	171	182	134	156	113	178	144	132	119
			102	177	126	123	185	115	233	128	138	188	250	124	172	173	108	181	172	153	120	179	188	134	166	113	178	146	132	135
	CBB cl2	19/B03; 28/B03	96	171	118	125	185	100	213	120	134	180	226	115	170	147	104	173	174	149	116	175	186	137	156	111	178	146	123	119
			108	177	120	127	185	102	233	132	138	180	246	118	170	173	104	177	174	151	120	183	190	137	166	113	178	146	126	131
			111	183	122	129												187												133
	Chaco23 col4	19/B06; 28/B06	108	175	121	117	185	102	243	114	130	192	236	121	170	163	106	177	168	151	120	171	192	134	156	111	178	148	126	127
			111	177	121	123	185	102	243	128	130	192	236	121	170	173	108	177	174	151	122	175	192	134	160	113	178	150	126	127
	Pot7b cl5	19/B04; 28/B04	102	177	120	117	183	102	219	114	134	178	234	121	170	165	108	177	166	153	116	171	182	134	156	113	178	144	132	119
			117	179	126	123	185	115	233	128	138	188	250	124	172	173	108	181	172	153	120	179	188	134	166	113	178	146	132	135
	IVV cl4	19/B05; 28/B05	96	177	118	125	185	100	213	120	130	180	226	115	170	173	104	173	174	149	116	183	186	137	156	111	178	146	123	119
			111	183	120	129	185	100	233	120	138	192	234	118	170	173	104	177	174	149	120	183	194	137	156	115	178	146	126	131
	Esm cl3	19/B04; 28/B04	102	177	120	117	183	102	219	114	134	178	234	121	170	165	108	177	166	153	116	171	182	134	156	113	178	144	132	119
			117	179	126	123	185	115	233	128	138	188	250	124	172	173	108	181	172	153	120	179	188	134	166	113	178	146	132	135
	Pot7a cl1	19/B04; 28/B04	102	177	120	117	183	102	219	114	134	178	234	121	170	165	108	177	166	153	116	171	182	134	156	113	178	144	132	119
			117	179	126	123	185	115	233	128	138	188	250	124	172	173	108	181	172	153	120	179	188	134	166	113	178	146	132	135
	T665 cl1	19/B07; 28/B07	96	175	120	117	185	102	213	130	128	178	228	118	170	173	104	177	172	\	116	179	194	134	170	113	178	146	129	119
			96	177	120	127	185	104	233	132	138	180	228	118	172	173	108	187	174	\	116	179	194	134	170	123	178	146	132	131
	Tu18 cl2	19/B01; 28/B01	96	177	118	125	185	100	213	120	130	180	226	115	170	173	104	173	174	149	116	183	186	137	156	111	178	146	123	119
			111	179	120	129	185	100	233	120	138	192	234	118	170	173	104	177	174	149	120	183	194	137	156	115	178	146	126	131
Core TcIII[b]	ARMA18 cl3	19/C04; 28/C04	105	185	114	117	203	102	254	138	124	180	216	94	172	153	104	177	170	145	116	177	186	125	146	109	174	138	114	125
			105	185	114	117	203	104	254	138	124	182	222	94	172	155	104	177	170	163	122	177	190	125	146	111	176	138	123	125
	ARMA13 cl1	19/C01; 28/C01	105	185	114	117	193	98	246	114	124	180	200	94	178	153	100	177	170	141	120	177	188	125	146	109	174	138	114	127
			105	185	114	117	203	98	285	136	130	182	220	94	180	155	104	177	170	145	120	177	194	125	146	111	176	138	114	127
	85/847 cl2	19/C08; 28/C08	105	183	114	117	191	104	254	114	130	174	206	97	176	153	100	177	170	149	116	177	188	125	146	107	176	138	114	123
			105	183	114	117	201	124	257	114	130	174	208	97	178	153	104	177	170	149	126	177	188	125	146	107	176	138	114	131
	M5631 cl5	19/C06; 28/C06	105	189	114	117	193	118	230	114	128	172	200	99	174	153	100	179	170	135	132	177	208	125	146	109	174	138	117	131
			105	189	114	117	193	118	230	114	128	172	232	99	174	153	100	179	170	137	132	177	214	125	146	109	176	138	117	131
	CM25 cl2	19/C05; 28/C05	105	177	114	117	197	100	269	126	130	186	228	97	172	153	104	177	168	149	110	175	190	119	146	109	178	138	114	131
			105	201	114	117	197	102	285	126	130	186	228	97	176	155	104	177	170	149	120	175	198	125	146	111	182	138	114	131
	JA2 cl2	19/C02; 28/C02	105	175	114	117	193	110	240	114	124	174	206	94	174	155	106	177	170	137	118	179	198	119	146	109	174	138	114	121
			105	195	114	117	195	110	269	128	124	176	206	97	174	155	106	177	170	137	122	181	198	125	146	111	176	138	114	133
	SABP19 cl1	19/C03; 28/C03	105	185	114	117	193	110	227	116	124	174	208	94	172	153	100	177	170	147	120	177	190	119	146	109	174	138	114	123
			105	189	114	117	193	122	264	130	128	180	212	97	180	155	100	177	170	147	122	177	192	125	146	109	176	138	123	127
	M6241 cl6	19/C07; 28/C07	105	209	114	117	185	118	269	126	130	176	208	94	174	155	104	177	170	135	108	175	208	119	146	109	174	138	111	121
			105	211	114	117	195	118	272	126	130	176	216	97	174	155	104	177	170	137	122	179	214	125	146	109	176	138	114	125
	X9/3	19/C09; 28/C09	105	185	114	117	193	102	235	130	130	180	200	94	178	153	100	177	170	145	116	177	186	125	146	111	174	138	114	123
			105	185	114	117	203	110	235	130	130	180	216	97	178	153	100	177	170	145	124	177	196	125	146	111	176	138	114	125
	X109/2	19/C10; 28/C10	105	185	114	117	193	110	242	116	124	174	208	97	172	153	100	177	170	147	120	177	190	125	146	109	176	138	114	123
			105	189	114	117	193	122	264	130	128	180	212	97	180	153	100	177	170	147	122	177	192	125	146	109	176	138	123	127

Core TcV[b]	PAH179 cl5	19/D02; 28/D02	105	177	114	117	185	100	227	128	124	180	210	97	170	153	100	177	170	145	120	177	188	125	146	105	178	138	114	125
			111	187	120	129	191	104	230	128	130	190	226	115	178	173	104	177	174	145	120	183	190	137	156	111	178	146	123	125
	Chaco2 cl3	19/D01; 28/D01	105	177	114	117	185	100	213	128	124	180	210	97	170	153	100	177	170	145	120	177	188	125	146	105	174	138	114	127
			111	187	120	129	191	104	230	128	130	192	226	115	178	173	104	177	174	145	120	183	190	137	156	111	178	146	123	127
	Para6 cl4	19/D01; 28/D01	105	177	114	117	185	100	213	128	124	180	210	97	170	153	100	177	170	145	120	177	188	125	146	105	174	138	114	127
			111	187	120	129	191	104	230	128	130	192	226	115	178	173	104	177	174	145	120	183	190	137	156	111	178	146	123	127
	Vinch101 cl1	19/D01; 28/D01	105	177	114	117	185	100	213	128	124	180	210	97	170	153	100	177	170	145	120	177	188	125	146	105	174	138	114	127
			111	187	120	129	191	104	230	128	130	192	226	115	178	173	104	177	174	145	120	183	190	137	156	111	178	146	123	127
	Sc43 cl1	19/D01; 28/D01	105	177	114	117	185	100	213	128	124	180	210	97	170	153	100	177	170	145	120	177	188	125	146	105	174	138	114	127
			111	187	120	129	191	104	230	128	130	192	226	115	178	173	104	177	174	145	120	183	190	137	156	111	178	146	123	127
	92-80 cl2	19/D01; 28/D01	105	177	114	117	185	100	213	128	124	180	210	97	170	153	100	177	170	145	120	177	188	125	146	105	174	138	114	127
			111	187	120	129	191	104	230	128	130	192	226	115	178	173	104	177	174	145	120	183	190	137	156	111	178	146	123	127
	Para4 cl3	19/D01; 28/D01	105	177	114	117	185	100	213	128	124	180	210	97	170	153	100	177	170	145	120	177	188	125	146	105	174	138	114	127
			111	187	120	129	191	104	230	128	130	192	226	115	178	173	104	177	174	145	120	183	190	137	156	111	178	146	123	127
	Bug2148 cl1	19/D01; 28/D01	105	177	114	117	185	100	213	128	124	180	210	97	170	153	100	177	170	145	120	177	188	125	146	105	174	138	114	127
			111	187	120	129	191	104	230	128	130	192	226	115	178	173	104	177	174	145	120	183	190	137	156	111	178	146	123	127
Core TcVI[b]	Chaco9 col15	19/E01; 28/E01	105	177	114	117	185	104	213	108	128	180	210	94	170	153	100	177	170	147	120	175	190	140	146	109	174	138	123	125
			111	187	120	129	195	122	254	130	136	180	262	115	178	173	106	187	172	147	120	177	190	140	170	117	178	148	138	125
	P251 cl7	19/E03; 28/E03	105	177	114	117	185	104	213	108	128	180	210	94	170	153	100	177	170	147	120	175	190	140	146	109	174	138	123	125
			111	187	120	129	195	122	254	130	136	180	258	115	178	173	106	187	172	147	120	177	190	140	170	117	178	148	138	125
																177														
	Chaco17 col1	19/E01; 28/E01	105	177	114	117	185	104	213	108	128	180	210	94	170	153	100	177	170	147	120	175	190	140	146	109	174	138	123	125
			111	187	120	129	195	122	254	130	136	180	262	115	178	173	106	187	172	147	120	177	190	140	170	117	178	148	138	125
	Tula cl2	19/E02; 28/E02	105	177	114	117	185	104	213	108	128	180	210	94	170	153	100	177	170	147	120	175	190	140	146	109	174	138	123	125
			111	187	120	129	195	122	254	130	136	180	258	115	178	173	106	187	172	147	120	177	190	140	170	117	178	148	138	125
	EPV20-1 cl1	19/E02; 28/E02	105	177	114	117	185	104	213	108	128	180	210	94	170	153	100	177	170	147	120	175	190	140	146	109	174	138	123	125
			111	187	120	129	195	122	254	130	136	180	258	115	178	173	106	187	172	147	120	177	190	140	170	117	178	148	138	125
	LHVA cl4	19/E01; 28/E01	105	177	114	117	185	104	213	108	128	180	210	94	170	153	100	177	170	147	120	175	190	140	146	109	174	138	123	125
			111	187	120	129	195	122	254	130	136	180	262	115	178	173	106	187	172	147	120	177	190	140	170	117	178	148	138	125
	CL Brener	19/E01; 28/E01	105	177	114	117	185	104	213	108	128	180	210	94	170	153	100	177	170	147	120	175	190	140	146	109	174	138	123	125
			111	187	120	129	195	122	254	130	136	180	262	115	178	173	106	187	172	147	120	177	190	140	170	117	178	148	138	125
	VFRA1 cl1	19/E01; 28/E01	105	177	114	117	185	104	213	108	128	180	210	94	170	153	100	177	170	147	120	175	190	140	146	109	174	138	123	125
			111	187	120	129	195	122	254	130	136	180	262	115	178	173	106	187	172	147	120	177	190	140	170	117	178	148	138	125
Suppl. TcI[c]	CJ007 (PI)	19/X06; ND	ND	167	ND	113	187	ND	ND	116	ND	ND	\	ND	\	159	104	179	178	ND	110	131	182	128	160	111	172	138	126	ND
			ND	167	ND	117	189	ND	ND	116	ND	ND	\	ND	\	163	106	179	186	ND	110	131	186	128	160	119	172	142	129	ND
	CJ005 (PII)	19/X05; ND	ND	167	ND	117	183	ND	ND	116	ND	ND	\	ND	\	161	104	179	170	ND	110	127	186	128	160	111	170	142	126	ND
			ND	167	ND	117	183	ND	ND	116	ND	ND	\	ND	\	163	104	179	176	ND	110	131	188	128	160	113	172	144	126	ND
	SAXP18 cl1	19/X01; ND	ND	167	ND	117	191	ND	ND	116	ND	ND	190	ND	170	159	104	179	172	ND	110	\	190	128	160	111	172	142	129	ND
			ND	167	ND	117	191	ND	ND	116	ND	ND	190	ND	176	159	104	179	172	ND	110	\	190	128	160	111	172	142	129	ND
	Chile C22 cl1	19/X02; ND	ND	167	ND	117	187	ND	ND	116	ND	ND	190	ND	178	159	104	179	172	ND	110	131	190	128	160	111	172	140	129	ND
			ND	167	ND	117	191	ND	ND	116	ND	ND	190	ND	178	159	104	179	172	ND	110	131	190	128	160	111	172	140	129	ND
	JR cl4	19/X04; ND	ND	167	ND	117	189	ND	ND	116	ND	ND	196	ND	170	163	104	\	176	ND	110	131	182	128	160	111	172	142	129	ND
			ND	169	ND	117	201	ND	ND	116	ND	ND	196	ND	170	163	104	\	176	ND	110	131	184	128	160	111	172	142	129	ND
	92101601P cl1	19/X03; ND	ND	167	ND	117	187	ND	ND	116	ND	ND	196	ND	172	159	104	185	170	ND	110	131	200	128	160	109	174	140	120	ND
			ND	167	ND	117	187	ND	ND	116	ND	ND	196	ND	176	159	104	185	172	ND	110	131	200	128	160	109	174	140	132	ND
	FLORIDA	19/X07; ND	ND	167	ND	117	187	ND	ND	116	ND	ND	\	ND	172	159	104	185	170	ND	110	131	200	128	160	109	174	140	120	ND

			ND	167	ND	117	187	ND	ND	116	ND	ND	\	ND	172	159	104	185	170	ND	110	131	200	128	160	109	174	140	132	ND
	M7	19/X08; ND	ND	\	ND	117	187	ND	ND	116	ND	ND	196	ND	172	163	104	185	172	ND	110	131	184	128	160	109	174	138	132	ND
			ND	\	ND	117	189	ND	ND	116	ND	ND	196	ND	172	163	104	185	174	ND	110	131	186	128	160	109	174	140	132	ND
	Xe1313 cl2	19/X09; ND	ND	167	ND	113	187	ND	ND	116	ND	ND	192	ND	176	159	104	179	178	ND	110	131	182	128	160	111	172	138	132	ND
			ND	167	ND	117	189	ND	ND	116	ND	ND	192	ND	176	159	106	179	186	ND	110	141	186	128	160	119	172	142	132	ND
	Xe3981	19/X10; ND	ND	167	ND	117	187	ND	ND	116	ND	ND	\	ND	170	163	104	179	\	ND	110	131	184	128	160	111	170	134	126	ND
			ND	167	ND	117	187	ND	ND	116	ND	ND	\	ND	170	163	106	177	\	ND	110	131	186	128	160	125	172	144	129	ND
	Sjm34 cl1	19/X11; ND	ND	167	ND	117	187	ND	ND	116	ND	ND	192	ND	182	163	104	179	176	ND	110	131	190	128	160	123	174	140	129	ND
			ND	167	ND	117	191	ND	ND	116	ND	ND	196	ND	182	163	104	179	184	ND	110	131	192	128	160	123	176	142	129	ND
	Xe5012	19/X12; ND	ND	169	ND	117	187	ND	ND	116	ND	ND	190	ND	172	163	104	177	170	ND	110	131	184	128	160	109	172	142	126	ND
			ND	169	ND	117	191	ND	ND	116	ND	ND	192	ND	174	165	104	177	176	ND	110	141	188	128	160	111	172	144	129	ND
	COTMA9	19/X13; ND	ND	167	ND	117	191	ND	ND	116	ND	ND	190	ND	170	155	104	\	172	ND	110	131	190	128	160	107	172	140	129	ND
			ND	167	ND	117	191	ND	ND	116	ND	ND	198	ND	170	159	104	\	172	ND	110	131	190	128	160	111	172	142	129	ND
	PALDA22	19/X14; ND	ND	167	ND	117	191	ND	ND	116	ND	ND	196	ND	174	155	104	194	176	ND	110	131	190	128	160	111	172	142	126	ND
			ND	167	ND	117	191	ND	ND	116	ND	ND	198	ND	174	163	104	194	182	ND	110	131	190	128	160	111	172	142	129	ND
	COTMA55	19/X15; ND	ND	167	ND	117	191	ND	ND	116	ND	ND	190	ND	170	157	104	179	172	ND	110	127	190	128	160	107	172	140	129	ND
			ND	167	ND	117	191	ND	ND	116	ND	ND	198	ND	170	159	104	179	172	ND	110	131	190	128	160	107	172	140	129	ND
	458 cl5	19/X16; ND	ND	167	ND	117	189	ND	ND	116	ND	ND	194	ND	176	157	104	179	170	ND	110	131	188	128	160	111	172	138	126	ND
			ND	167	ND	117	189	ND	ND	116	ND	ND	194	ND	176	163	104	179	178	ND	110	131	190	128	160	113	172	140	132	ND
	PALDA3	19/X17; ND	ND	167	ND	117	191	ND	ND	116	ND	ND	196	ND	\	155	104	194	176	ND	110	131	188	128	160	113	170	140	129	ND
			ND	167	ND	117	191	ND	ND	116	ND	ND	196	ND	\	163	104	194	182	ND	110	131	188	128	160	113	176	142	129	ND
	TEDA	19/X18; ND	ND	167	ND	117	191	ND	ND	116	ND	ND	196	ND	174	155	104	194	176	ND	110	131	190	128	160	111	172	142	126	ND
			ND	167	ND	117	191	ND	ND	116	ND	ND	198	ND	174	163	104	194	182	ND	110	131	190	128	160	111	176	142	129	ND
	361ta	19/X19; ND	ND	169	ND	\	189	ND	ND	116	ND	ND	198	ND	172	155	104	185	172	ND	110	131	188	128	160	109	174	140	126	ND
			ND	169	ND	\	189	ND	ND	116	ND	ND	198	ND	172	155	104	185	172	ND	110	131	188	128	160	109	174	140	126	ND
	M13 cl3	19/X20; ND	ND	167	ND	117	187	ND	ND	116	ND	ND	194	ND	172	163	104	\	166	ND	110	131	182	128	160	109	172	138	126	ND
			ND	167	ND	117	201	ND	ND	116	ND	ND	198	ND	176	163	104	\	182	ND	110	131	182	128	160	109	172	140	129	ND
	M18 cl4	19/X21; ND	ND	167	ND	117	187	ND	ND	116	ND	ND	196	ND	172	163	104	189	166	ND	110	131	182	128	160	109	174	138	129	ND
			ND	169	ND	117	189	ND	ND	116	ND	ND	198	ND	178	163	106	189	172	ND	110	131	192	128	160	111	174	142	132	ND
	93070103P	19/X07; ND	ND	167	ND	117	187	ND	ND	116	ND	ND	196	ND	172	159	104	185	170	ND	110	131	200	128	160	109	174	140	120	ND
			ND	167	ND	117	187	ND	ND	116	ND	ND	196	ND	172	159	104	185	170	ND	110	131	200	128	160	109	174	140	132	ND
	PALDA5	19/X23; ND	ND	167	ND	117	191	ND	ND	116	ND	ND	190	ND	176	155	104	194	182	ND	110	131	190	128	160	111	172	140	126	ND
			ND	167	ND	117	191	ND	ND	116	ND	ND	198	ND	176	159	104	194	182	ND	110	131	190	128	160	111	172	140	129	ND
	Sjm32	19/X24; ND	ND	167	ND	117	193	ND	ND	116	ND	ND	190	ND	168	159	104	\	172	ND	110	131	182	128	160	109	172	132	126	ND
			ND	167	ND	117	195	ND	ND	116	ND	ND	190	ND	168	163	104	\	172	ND	110	131	186	128	160	109	174	142	126	ND
	Sjmc7	19/X25; ND	ND	167	ND	117	203	ND	ND	116	ND	ND	\	ND	168	159	104	177	172	ND	110	131	188	128	160	109	172	132	126	ND
			ND	167	ND	117	203	ND	ND	116	ND	ND	\	ND	168	159	104	185	172	ND	110	131	190	128	160	109	172	140	126	ND
	Xe5165	19/X26; ND	ND	167	ND	117	187	ND	ND	116	ND	ND	194	ND	170	159	104	183	178	ND	110	131	182	128	160	111	172	140	132	ND
			ND	167	ND	117	195	ND	ND	116	ND	ND	194	ND	180	159	104	183	178	ND	110	131	188	128	160	115	172	142	132	ND
	USA Opossum cl2	19/X27; ND	ND	167	ND	117	187	ND	ND	116	ND	ND	196	ND	172	159	104	185	170	ND	110	131	184	128	160	109	174	140	132	ND
			ND	167	ND	117	187	ND	ND	116	ND	ND	196	ND	172	159	104	185	170	ND	110	131	202	128	160	109	174	140	132	ND
	USA Arma cl1	19/X22; ND	ND	167	ND	117	187	ND	ND	116	ND	ND	196	ND	172	159	104	185	170	ND	110	131	184	128	160	109	174	140	132	ND
			ND	167	ND	117	187	ND	ND	116	ND	ND	196	ND	174	159	104	185	170	ND	110	131	184	128	160	109	174	140	132	ND
Suppl. TcIII[d]	CM17	19/C11; ND	ND	169	ND	117	185	ND	ND	114	ND	ND	204	ND	174	155	106	177	170	ND	108	179	198	125	146	109	176	138	114	ND
			ND	169	ND	117	195	ND	ND	126	ND	ND	210	ND	174	155	106	177	170	ND	114	179	212	125	146	109	176	138	114	ND
	m10	19/C12; ND	ND	187	ND	117	195	ND	ND	114	ND	ND	206	ND	174	151	104	177	170	ND	118	181	196	125	146	109	176	138	114	ND

			ND	187	ND	117	197	ND	ND	130	ND	ND	210	ND	176	155	106	177	170	ND	120	183	212	125	146	109	178	138	114	ND
	cayma19	19/C13; ND	ND	187	ND	117	203	ND	ND	136	ND	ND	206	ND	178	153	104	177	170	ND	120	177	186	125	146	107	176	138	114	ND
			ND	187	ND	117	205	ND	ND	138	ND	ND	212	ND	178	153	104	177	170	ND	120	177	192	125	146	109	176	138	114	ND
	Ma194	19/C14; ND	ND	185	ND	117	195	ND	ND	130	ND	ND	\	ND	176	153	100	177	168	ND	108	177	188	125	146	109	174	138	114	ND
			ND	189	ND	117	195	ND	ND	130	ND	ND	\	ND	178	153	100	177	168	ND	124	177	190	125	146	109	176	138	114	ND
	Sp4	19/C15; ND	ND	185	ND	117	193	ND	ND	130	ND	ND	200	ND	172	153	100	177	170	ND	108	177	188	125	146	111	174	138	114	ND
			ND	189	ND	117	195	ND	ND	138	ND	ND	200	ND	176	153	100	177	170	ND	116	177	192	125	146	111	174	138	123	ND
	Sjmc4	19/C16; ND	ND	187	ND	117	203	ND	ND	130	ND	ND	\	ND	178	153	104	177	170	ND	122	177	188	125	146	107	174	138	114	ND
			ND	189	ND	117	203	ND	ND	134	ND	ND	\	ND	180	153	104	177	170	ND	122	177	188	125	146	113	176	138	114	ND
	Sjmc10	19/C17; ND	ND	185	ND	117	201	ND	ND	112	ND	ND	\	ND	172	153	104	177	170	ND	122	177	188	125	146	113	174	138	114	ND
			ND	185	ND	117	203	ND	ND	122	ND	ND	\	ND	176	155	104	177	170	ND	124	177	188	125	146	113	174	138	114	ND
	Sam6	19/C18; ND	ND	187	ND	117	193	ND	ND	130	ND	ND	210	ND	164	153	104	177	170	ND	108	177	192	125	146	105	174	138	114	ND
			ND	187	ND	117	203	ND	ND	142	ND	ND	212	ND	172	155	104	177	170	ND	108	177	192	125	146	109	174	138	123	ND
	cayma14	19/C19; ND	ND	185	ND	117	195	ND	ND	112	ND	ND	\	ND	172	155	104	177	170	ND	108	177	186	125	146	105	\	138	114	ND
			ND	187	ND	117	209	ND	ND	130	ND	ND	\	ND	178	155	104	177	170	ND	120	177	192	125	146	109	\	138	114	ND
	Arma12	19/C20; ND	ND	185	ND	117	193	ND	ND	134	ND	ND	214	ND	172	153	100	177	170	ND	108	177	194	125	146	105	174	124	114	ND
			ND	187	ND	117	203	ND	ND	136	ND	ND	218	ND	176	155	104	177	170	ND	122	177	200	125	146	109	174	138	114	ND
	Arma9	19/C21; ND	ND	185	ND	117	195	ND	ND	130	ND	ND	206	ND	\	153	100	177	170	ND	122	177	186	125	146	109	174	138	114	ND
			ND	187	ND	117	195	ND	ND	132	ND	ND	210	ND	\	157	100	177	170	ND	122	177	192	125	146	109	174	138	123	ND
	Cayma18	19/C22; ND	ND	187	ND	117	\	ND	ND	114	ND	ND	204	ND	\	155	104	177	170	ND	120	177	194	125	146	107	176	138	114	ND
			ND	187	ND	117	\	ND	ND	114	ND	ND	210	ND	\	155	104	177	170	ND	122	179	194	125	146	107	176	138	123	ND
	Sp13	19/C23; ND	ND	185	ND	117	193	ND	ND	130	ND	ND	200	ND	172	153	100	177	170	ND	118	177	190	125	146	109	174	138	114	ND
			ND	189	ND	117	195	ND	ND	130	ND	ND	206	ND	178	153	100	177	170	ND	124	179	194	125	146	111	176	138	114	ND
	Sp15	19/C24; ND	ND	185	ND	117	193	ND	ND	130	ND	ND	200	ND	172	153	100	177	170	ND	118	177	190	125	146	109	174	138	114	ND
			ND	185	ND	117	195	ND	ND	130	ND	ND	206	ND	178	153	100	177	170	ND	124	179	194	125	146	111	176	138	114	ND
	Sp16	19/C23; ND	ND	185	ND	117	193	ND	ND	130	ND	ND	200	ND	172	153	100	177	170	ND	118	177	190	125	146	109	174	138	114	ND
			ND	189	ND	117	195	ND	ND	130	ND	ND	206	ND	178	153	100	177	170	ND	124	179	194	125	146	111	176	138	114	ND
Suppl. TcIV	10R26	19/A02; ND	ND	173	ND	125	185	ND	ND	110	ND	ND	222	ND	172	153	104	189	180	ND	112	155	190	125	148	\	174	152	132	ND
			ND	177	ND	133	185	ND	ND	114	ND	ND	224	ND	172	153	104	189	180	ND	112	155	190	128	148	\	174	154	132	ND
	ERA cl2	19/A03; ND	ND	179	ND	123	187	ND	ND	114	ND	ND	198	ND	170	153	104	187	180	ND	106	163	\	128	148	107	180	152	132	ND
			ND	179	ND	123	187	ND	ND	114	ND	ND	198	ND	170	153	104	187	180	ND	112	169	\	128	148	107	180	152	132	ND
	CanIII cl1	19/A01; ND	ND	173	ND	123	191	ND	ND	114	ND	ND	196	ND	170	155	106	183	178	ND	106	167	\	131	162	109	174	144	132	ND
			ND	173	ND	123	191	ND	ND	114	ND	ND	196	ND	170	155	106	185	178	ND	112	167	\	131	162	109	174	144	132	ND

ND Not determined

\ Missing data (no amplification or ambiguous profile)

[a] Multilocus genotype

[b] Data for 8 loci (MCLF10, 10101(TA), 6925(TG)a, 11283(TCG), 7093(TC), 10101(TC), 6925(CT), 7093(TCC)) previously published [1]

[c] Data previously published [2]

[d] Data previously published [3]

1. Lewis MD, Llewellyn MS, Gaunt MW, Yeo M, Carrasco HJ, et al. (2009) Flow cytometric analysis and microsatellite genotyping reveal extensive DNA content variation in *Trypanosoma cruzi* populations and expose contrasts between natural and experimental hybrids. Int J Parasitol 39: 1305-1317.
2. Llewellyn MS, Miles MA, Carrasco HJ, Lewis MD, Yeo M, et al. (2009) Genome-scale multilocus microsatellite typing of *Trypanosoma cruzi* discrete typing unit I reveals phylogeographic structure and specific genotypes linked to human infection. PLoS Pathog 5: e1000410.
3. Llewellyn MS, Lewis MD, Acosta N, Yeo M, Carrasco HJ, et al. (2009) *Trypanosoma cruzi* IIc: Phylogenetic and Phylogeographic Insights from Sequence and Microsatellite Analysis and Potential Impact on Emergent Chagas Disease. PLoS Negl Trop Dis 3: e510.
